# Targeting Cullin–RING E3 ubiquitin ligases for drug discovery: structure, assembly and small-molecule modulation

**DOI:** 10.1042/BJ20141450

**Published:** 2015-04-17

**Authors:** Emil Bulatov, Alessio Ciulli

**Affiliations:** *College of Life Sciences, University of Dundee, Dundee DD1 5EH, U.K.; †Department of Chemistry, University of Cambridge, Cambridge CB2 1EW, U.K.

**Keywords:** assembly, small molecule, structure, structure-based design, ubiquitin, ubiquitination, APC/C, anaphase-promoting complex/cyclosome, ASB, ankyrin repeat and SOCS-box, Aux, auxin, BCR, BTB–Cul3–Rbx1, BP, β-propeller, BTB, bric-a-brac/tramtrack/broad complex, CAND1, Cullin-associated NEDD8-dissociated protein 1, CBFβ, core binding factor β, Cdc, cell division cycle, Cks1, cyclin-dependent protein kinase regulatory subunit 1, COI1, coronatine-insensitive protein 1, Cpd, compound, CPH, conserved within Cul7, PARC and HERC2, CRBN, cereblon, CRL, Cullin–RING E3 ubiquitin ligase, CSA, Cockayne syndrome A, CSN, COP9 (constitutive photomorphogenesis 9) signalosome complex, CTD, C-terminal domain, Cul, Cullin, DCAF, DDB1–Cul4A-associated factor, DDB, damage-specific DNA-binding protein, EloBC, ElonginB–ElonginC complex, Fbw/Fbxw, F-box/WD repeat-containing protein, Fbxl, F-box/leucine-rich motif-containing protein, Fbxo, F-box/other domain-containing protein, FP, fluorescence polarization, GHR, growth hormone receptor, HECT, homologous with E6-associated protein C-terminus, HERC2, HECT domain- and RLD (regulator of chromosome condensation 1 protein-like domain) domain-containing E3 ubiquitin protein ligase 2, HIF-1α, hypoxia-inducible factor 1α, IAA, indole-3-acetic acid, IκB, inhibitor of NF-κB, ITC, isothermal titration calorimetry, JA-Ile, jasmonoyl-isoleucine, JAZ1, jasmonate/ZIM (zinc finger expressed in inflorescence) domain protein 1, Keap1, Kelch-like enoyl-CoA hydratase-associated protein 1, KLHL, Kelch-like protein, MATH, meprin and TRAF (tumour necrosis factor receptor-associated factor) homology, MEL26, maternal effect lethal 26, mTOR, mammalian target of rapamycin, NAE, NEDD8-activating enzyme, NEDD, neural-precursor-cell-expressed developmentally down-regulated, NF-κB, nuclear factor κB, Nrf2, nuclear factor-erythroid 2-related factor 2, NTD, N-terminal domain, PARC, p53-associated parkin-like cytoplasmic protein, POZ, pox virus and zinc finger, PPI, protein–protein interaction, Protac, proteolysis-targeting chimaeric molecule, Rbx1, RING-box protein 1, RING, really interesting new gene, SCF, Skp1–Cdc53–F-box Cdc4, SH2, Src homology 2, Skp2, S-phase kinase-associated protein 2, SMER3, small-molecule enhancer of rapamycin 3, SOCS, suppressor of cytokine signalling, SPOP, speckle-type POZ protein, STAT, signal transducer and activator of transcription, SV5, simian virus 5, TIR1, transport inhibitor response 1, β-TrCP, β-transducin repeat-containing protein, Ub, ubiquitin, Ubc12, ubiquitin-conjugating enzyme 12, UBL, ubiquitin-like protein, UPS, ubiquitin–proteasome system, VHL, von Hippel–Lindau, Vif, virion infectivity factor, Vpr, viral protein R, VPRBP, Vpr-binding protein

## Abstract

In the last decade, the ubiquitin–proteasome system has emerged as a valid target for the development of novel therapeutics. E3 ubiquitin ligases are particularly attractive targets because they confer substrate specificity on the ubiquitin system. CRLs [Cullin–RING (really interesting new gene) E3 ubiquitin ligases] draw particular attention, being the largest family of E3s. The CRLs assemble into functional multisubunit complexes using a repertoire of substrate receptors, adaptors, Cullin scaffolds and RING-box proteins. Drug discovery targeting CRLs is growing in importance due to mounting evidence pointing to significant roles of these enzymes in diverse biological processes and human diseases, including cancer, where CRLs and their substrates often function as tumour suppressors or oncogenes. In the present review, we provide an account of the assembly and structure of CRL complexes, and outline the current state of the field in terms of available knowledge of small-molecule inhibitors and modulators of CRL activity. A comprehensive overview of the reported crystal structures of CRL subunits, components and full-size complexes, alone or with bound small molecules and substrate peptides, is included. This information is providing increasing opportunities to aid the rational structure-based design of chemical probes and potential small-molecule therapeutics targeting CRLs.

## INTRODUCTION

In the last decade the field of the UPS (ubiquitin–proteasome system) has witnessed increasing attention of the scientific community, especially as a result of the award of the 2004 Nobel Prize in Chemistry to Aaron Ciechanover, Avram Hershko and Irwin Rose for the discovery of ubiquitin-mediated protein degradation [1]. The breakthrough discovery highlighted the importance of studies in this area and promoted substantial funding allocation to support research on the subject. The overall mechanism of the ubiquitin–proteasome pathway is regulated by the sequential action of three enzymes (E1–E2–E3) [2,3]. The human genome encodes two E1-activating enzymes, 37 E2-conjugating enzymes and over 600 E3 ubiquitin ligases [4]. First, a ubiquitin molecule is chemically activated in an ATP-dependent manner by an E1-activating enzyme forming a thioester bond between the C-terminal glycine residue of ubiquitin and a conserved cysteine residue of the E1. Next, ubiquitin is transferred on to an E2-conjugating enzyme via a *trans*-thiolation reaction. Finally, an isopeptide bond between the ε-amino group of a substrate lysine residue and the C-terminal glycine residue of ubiquitin is formed via E3 ligase-mediated catalysis, and then between Ub molecules to form poly-Ub chains. As a result of this three-step conjugating cascade, ubiquitinated substrates can be recognized and degraded by the 26S proteasome in an ATP-dependent manner or downstream cell signalling responses are triggered [5,6]. Protein ubiquitination is reversible and the isopeptide bond can be hydrolysed by protease enzymes called deubiquitinases (DUBs), of which over 80 have been identified in the human genome [7].

The UPS is growing in importance as a therapeutic target as a result of being increasingly linked to human diseases, including cancer, diabetes and inflammation [8–10]. Despite demonstrated clinical and commercial success of proteasome inhibitors, several issues remain to be addressed with targeting the proteasome therapeutically, including broad cellular impact, potential risk of side effects and increasing resistance. An attractive alternative approach would be targeting enzymes upstream of the proteasome, particularly E3 ligases that confer substrate selectivity on the UPS. The CRLs [Cullin–RING (really interesting new gene) E3 ubiquitin ligases] constitute the main family of E3s that signal substrates to proteasomal degradation and represent promising therapeutic targets. The growing number of solved CRL crystal structures is improving our understanding of the assembly and function of these enzymes, providing more opportunities for the rational structure-based design of small-molecule inhibitors and modulators.

In the present review, we focus on current knowledge concerning the structure and assembly of CRL complexes. We then highlight recent efforts at identifying small-molecule ligands, and discuss opportunities and challenges of targeting these enzymes for drug discovery.

## TYPES OF E3 LIGASES

The E3 ubiquitin ligases can be divided into two major families on the basis of their assembly and mechanism of action: the HECT (homologous with E6-associated protein C-terminus) domain and the RING domain. HECT E3s accept ubiquitin (Ub) from E2~Ub to form a covalent thioester intermediate via a conserved cysteine residue of the E3 itself before transferring ubiquitin on to the substrate. In contrast, RING E3s directly transfer ubiquitin to the substrate by bringing both E2~Ub and the substrate in close proximity to each other. The RBR (RING–between RING–RING) ligases represent an additional family of E3s that combine characteristics of both HECT and RING families, as they recruit E2~Ub conjugates by an N-terminal RING domain and then transfer ubiquitin on to a HECT-type C-terminal catalytic cysteine residue of the E3 before final transfer on to the substrate [11].

The RING domain was originally discovered in the protein Ring1. Later, four independent groups established that the RING protein Rbx1 (RING-box protein 1) serves as a CRL1 subunit that recruits an E2 enzyme [12–15]. The distinctive feature of RING E3s is a canonical structural motif that co-ordinates two Zn^2+^ ions, Cys-Xaa_2_-Cys-Xaa_9–39_-Cys-Xaa_1–3_-His-Xaa_2–3_-Cys-Xaa_2_-Cys-Xaa_4–48_-Cys-Xaa_2_-Cys (Xaa is any amino acid) [16]. RING E3s constitute the largest superfamily of E3 ligases that can be further categorized into two subgroups: CRLs and APC/C (anaphase-promoting complex/cyclosome) [17].

Both CRL and APC/C are multisubunit complexes and share some compositional resemblance. For example, they are both constituted of adaptors and substrate receptor subunits, e.g. F-box proteins for CRL [Skp2 (S-phase kinase-associated protein 2), Fbw7 (F-box and WD repeat domain-containing 7), β-TrCP (β-transducin repeat-containing protein)] and activators for APC/C [Cdc20 (cell division cycle 20) and Cdh1 (Cdc20 homologue 1)]. Although APC/C and PARC (p53-associated parkin-like cytoplasmic protein) proteins share a degree of structural similarity with CRLs, they will not be covered here.

## CULLIN RING E3 UBIQUITIN LIGASES AND THEIR STRUCTURAL ORGANIZATION

**Figure 1:**
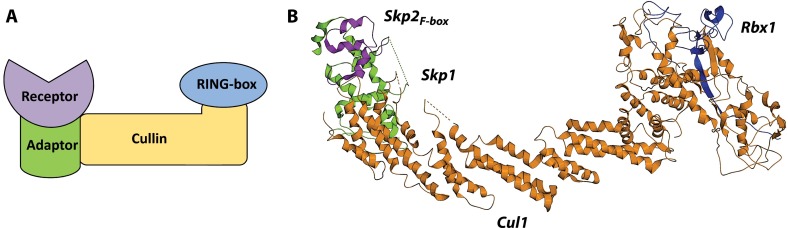
Cullin RING E3 ubiquitin ligases (**A**) General subunit organization of E3 CRLs showing receptor, adaptor, Cullin scaffold and Rbx RING-box subunits. (**B**) The crystal structure of the canonical CRL1^Skp2^ complex with F-box protein Skp2 as a substrate receptor (PDB code 1LDK).

The CRLs comprise over 200 members, making it the largest family of all E3s [18]. In some cell types, up to 20% of the proteasome-dependent degradation of the proteome is mediated by CRLs [19]. The evolutionarily conserved Cullin family has seven key members that share similar structural architecture: Cul1, Cul2, Cul3, Cul4A, Cul4B, Cul5 and Cul7. The classification of CRLs is based on the type of Cullin protein in the complex. This includes abbreviations such as SCF (Skp1–Cdc53–F-box Cdc4) [20], ECS (EloBC–Cul2/5–SOCS-box, where EloBC is ElonginB–ElonginC complex and SOCS is suppressor of cytokine signalling) [21] and BCR (BTB–Cul3–Rbx1, where BTB is bric-a-brac/tramtrack/broad complex) [22]. For clarity in the present review, we consistently use the CRL1–CRL7 abbreviations for CRL E3 ligases containing Cul1–Cul7 scaffold proteins respectively. CRLs use modular subunit organization by utilizing interchangeable adaptors, receptors, Cullin scaffolds and RING-box domains to enable assembly of a large number of functionally diverse E3 ligase complexes (Figure 1 and Table 1). The elongated Cullin NTD (N-terminal domain) consists of three so-called ‘Cullin repeats’, each formed by five α-helices, and recruits different substrate receptors either directly or via an adaptor subunit. The interaction between the adaptor–receptor complex and the Cullin NTD is often very tight and constitutes an important structural element of the CRL assembly. The structural features of each interface direct the selection of the correct subunits. Of particular importance is a so-called LPXP motif within the receptor subunit that forms a minor yet crucial supplementary interaction with the Cullin NTD [21]. Examples of adaptor proteins utilized in CRL assembly include Skp1, EloBC binary complex, BTB and DDB1 (damage-specific DNA-binding protein 1). Notably, Skp1, ElonginC and BTB display a high level of structural homology with each other and share a common fold that is often termed the Skp1/BTB/POZ fold (POZ is pox virus and zinc finger) [23]. In contrast, DDB1 differs from other adaptors in that it is considerably larger in size and consists of multiple distinctive β-propeller motifs.

**Table 1:** Subunit organization within the CRL family classified on the basis of the type of Cullins Included are information on adaptor, substrate receptor and Rbx RING protein for each of the respective Cul1–Cul7 scaffolds. A schematic representation for each of the CRLs is provided.

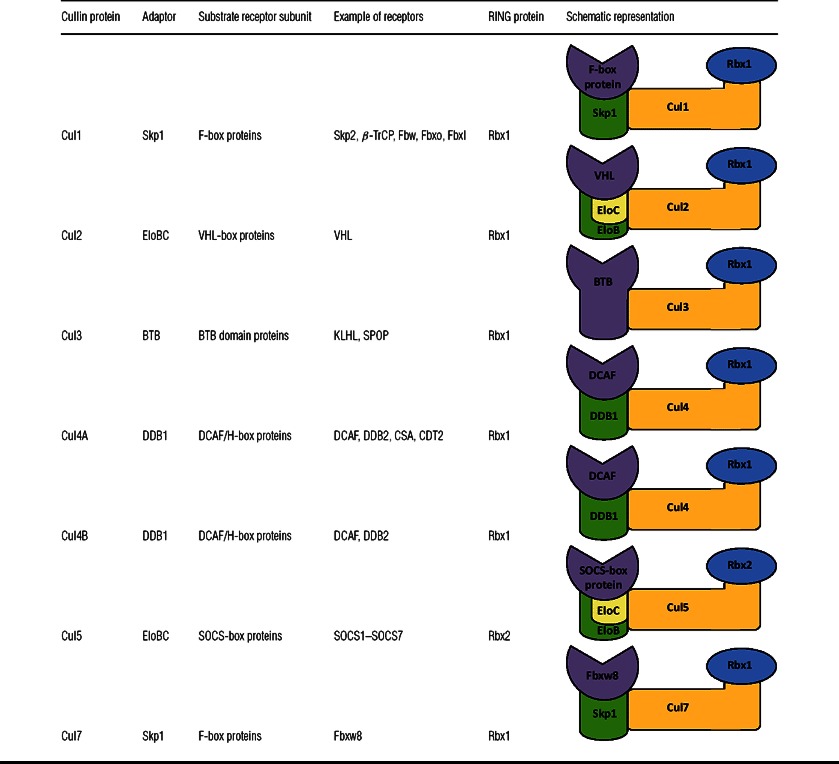

Specificity of CRL activity is determined by substrate-specific receptors that often function through selective recognition of post-translational modifications in the substrates, including phosphorylation and hydroxylation. Each receptor can potentially bind multiple different substrates, therefore expanding the functional range of CRL activity. Substrate receptors either consist of an individual subunit, which typically recruits the adaptor subunit(s) via an F-box, VHL (von Hippel–Lindau)/SOCS-box or H-box domain, or can be merged with the adaptor in a single polypeptide chain, i.e. BTB proteins (Table 1).

The conserved globular CTD (C-terminal domain) of Cullin serves as a docking site for RING-box proteins, such as Rbx1 or Rbx2. These proteins recruit a cognate ubiquitin-loaded E2 enzyme and subsequently promote the discharge of ubiquitin from E2 directly on to a substrate. The interaction between E2 enzyme and RING E3s is generally weak [24], but activity of the E3 ligase does not necessarily correlate with the stability of an E3–E2 association. Often, the physiological E2 partner of a particular E3 ligase is not known and empirical approaches are necessary to identify it [16].

The individual members of the Cullin family will now be described, with a particular focus on their assembly, structural features and biological functions.

### CRL1

The *Cul1* gene (also known as *Cdc53*) was originally discovered in *Caenorhabditis elegans* and budding yeast [25,26] and is therefore considered the founding member of the family. Subsequently, the archetypal example of CRL1, the protein complex Skp1–Cdc53–F-box Cdc4, was characterized in yeast [20]. In this complex, adaptor Skp1, substrate receptor Cdc4, and scaffold Cdc53 (a yeast orthologue of Cul1) assemble together to form the E3 ligase.

The high variety of receptor subunits within the CRL machinery allows functional diversity and targeting of different substrates. Generally, the NTD of the F-box proteins binds the adaptor subunit and the C-terminal part recruits in the substrate. The 69 F-box proteins reported to date are divided into three subgroups according to the structural feature of their substrate-binding domain: 12 Fbxw proteins (containing a WD40 domain), 21 Fbxl proteins (leucine-rich motifs) and 36 Fbxo proteins (other domains) [27]. The structural basis of substrate recognition is mainly determined by post-translational modification of short epitopes (degrons) of the substrate, e.g. phosphorylation, hydroxylation or glycosylation [28].

**Figure 2:**
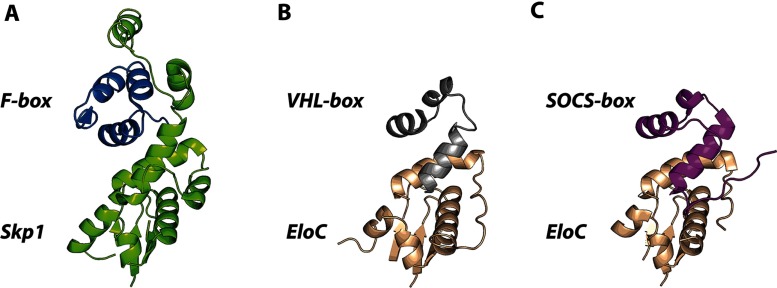
Assembly between substrate receptor ‘box’ domains and adaptor subunits (**A**) F-box domain of receptor Skp2 in complex with adaptor Skp1 (PDB code 2ASS). (**B**) VHL-box domain of receptor VHL in complex with adaptor subunit ElonginC (PDB code 1VCB). (**C**) SOCS-box domain of receptor SOCS2 in complex with ElonginC (PDB code 2C9W). The SOCS-box and VHL-box domains possess a high degree of structural similarity when complexed with ElonginC. The adaptor subunits Skp1 and ElonginC are structurally homologous proteins that form conserved binding interfaces with the N-terminal H1 helix of F-box and the C-terminal H3 helix of VHL-box/SOCS-box respectively.

One of the most studied members of CRL1 is constituted by the Cullin scaffold bridging the RING-box protein Rbx1 and the adaptor Skp1 bound to substrate receptor Skp2. The Cul1_NTD_ region that interacts with adaptor Skp1 is highly conserved in different species, but not within the Cullin family. The NTD of Skp1 interacts with Cul1, whereas its CTD binds the F-box motif of the substrate receptor subunit (Figure 2A). The crystal structure of Skp2_F-box_–Skp1–Cul1–Rbx1 complex provided the first structural information on a full-length Cullin scaffold and its interactions with other components of a CRL complex [29] (Figure 1B).

The crucial role of CRL1^Skp2^ in many cancers was firmly established in cellular and animal model studies. Skp2 is an oncoprotein that is overexpressed in many cancers [30]. CRL1^Skp2^ specifically recognizes phosphorylated p27^Kip1^, which is its best-characterized substrate to date. Skp2-dependent decrease in p27 levels was observed in cancer cells, leading to poor prognosis [31]. Disrupting the Skp2-p27 interaction using small molecules should inhibit p27 ubiquitination, resulting in an increase in p27 protein levels. In turn, this would be expected to reduce cancer cells proliferation, therefore providing an attractive therapeutic strategy.

In general, substrate ubiquitination depends solely on a fully formed and active CRL. However, in some cases, alternative factors may be required. It has been demonstrated that ubiquitination of p27 by CRL1^Skp2^ requires the accessory protein Cks1 (cyclin-dependent protein kinase regulatory subunit 1) that interacts with Skp2 [32]. The crystal structure of Skp1–Skp2–Cks1 in complex with a p27 phosphopeptide revealed that two residues of p27 form key contacts with both Cks1 and Skp2 proteins: Thr^187^, which interacts with the phosphate specific domains of Cks1, and Glu^185^, which is buried at the Cks1–Skp2 interface [33]. In addition, substrate-assisted assembly was recently reported for the Fbxl3–Skp1–Cul1 complex, which requires substrate Cry1 for the *in vivo* formation of CRL1^Fbxl3^ [34].

The wide range of receptor subunits and their targeted substrates mean that CRL1s play crucial roles in numerous cellular processes and physiological dysfunctions. Several prominent F-box substrate receptors of CRL1, such as Skp2, β-TrCP, Fbw7 and Fbxl3, were shown to play significant roles in cancer and other diseases [28]. Substrates of F-box proteins include protein kinases, cyclins and many other factors involved in different cellular processes, such as growth, signalling, differentiation and development, as reviewed in [27]. Another important member of the CRL1 subfamily, CRL1^β-TrCP^, demonstrates oncogenic properties mainly due to overexpression of β-TrCP in different types of cancer [35]. The β-TrCP receptor functions in the NF-κB (nuclear factor κB) signalling pathway by ubiquitinating IκB (inhibitor of NF-κB) that suppresses the NF-κB nuclear localization signal and inhibits its interaction with DNA [36]. The substrate-binding domain of β-TrCP comprises a β-propeller fold formed by seven WD40 repeats that recognizes phosphorylated degron motifs of substrates [37]. Moreover, β-TrCP targets β-catenin, DEPTOR [DEP domain-containing mTOR (mammalian target of rapamycin)-interacting protein] and other substrates for proteasomal degradation resulting in inhibition of their signalling pathways, thus playing key roles in cancer and inflammatory processes [27]. Overall, there is a strong rationale and solid scientific basis for the structure-based design of potent CRL1 inhibitors given that these enzymes are structurally well characterized.

### CRL2 AND CRL5

Other members of the Cullin family employ similar subunit architecture to that of the prototypical CRL1. Both Cul2 and Cul5 utilize the same EloBC adaptor complex for the assembly of functional CRL machineries. ElonginB and ElonginC proteins were originally discovered in a complex with ElonginA that acts to enhance the rate of RNA polymerase II elongation [38]. The EloBC adaptor binds to the so-called SOCS-box domain of the substrate receptor (Figures 2B and 2C). The broad class of SOCS-box proteins inherit their name from the SOCS proteins, one of the key substrate receptor classes for CRL5 that negatively regulate the JAK (Janus kinase)/STAT (signal transducer and activator of transcription) signal transduction pathway and perform important functions in the immune response [39]. Interestingly, the motif of ElonginA that is responsible for the recruitment of EloBC is highly homologous with the SOCS-box.

Over 80 SOCS-box proteins have been reported to date, including subclasses of proteins such as SOCS, VHL, ASB (ankyrin repeat and SOCS-box), ElonginA, WSB (WD repeat and SOCS-box) among others [40–42]. The SOCS-box proteins recruit EloBC, Cullin and RING-box protein to form the E3 ligase. The selection of Cullin scaffold occurs via the LPXP motif within the so-called ‘Cullin box’ at the C-terminal end of the SOCS-box [21]. A crystal structure of Cul2 in complex with EloBC and a substrate receptor (e.g. VHL) would help in elucidating the structural basis of selectivity for Cul5 rather than Cul2, but has yet remained elusive (see Note added in proof).

The interface between SOCS-box domains and EloBC is governed by hydrophobic interactions. The burial of hydrophobic patches in part explains why EloBC complex is required for stabilization of the otherwise disordered SOCS-box domain of receptor proteins [43]. This is supported further by the observation that the crystal structures of the full-size SOCS-box proteins have to date been solved only in complex with EloBC, i.e. SOCS2–EloBC [44], SOCS4–EloBC [45], SOCS2–EloBC–Cul5_NTD_ [46] and VHL–EloBC [47]. The majority of SOCS-box proteins recruit Cul5–Rbx2 to constitute the CRL5 E3 ligases. However, owing to minor differences in the Cullin box motif, some proteins such as VHL engage Cul2–Rbx1 instead to form CRL2 complexes [48]. Substrate receptors of CRL2 therefore belong to a different class termed VHL-box proteins.

The SOCS proteins consist of eight structurally homologous members, SOCS1–SOCS7 and CIS [cytokine-inducible SH2 (Src homology 2)-containing protein], that share a conserved C-terminal SOCS-box motif and a central SH2 domain. Analogously to when present in protein kinases, the SH2 domain recruits phosphotyrosine-containing sequences of the substrate protein. The SOCS proteins perform important roles in the immune response and cancer [39,49]. For example, the SOCS2 member negatively regulates growth hormone signalling by targeting GHR (growth hormone receptor) for ubiquitination and proteasomal degradation [44]. A recent study has showed that all components of the CRL5^SOCS2^ complex [SOCS2, EloBC, Cul5, Rbx2 and NEDD8 (neural-precursor-cell-expressed developmentally down-regulated 8)] can be recruited from human cell lysates using phosphorylated GHR peptides [50]. The recombinant full-size complex was reconstituted *in vitro* from individual components and the assembly and PPIs (protein–protein interactions) of complexes of different sizes, including NEDDylated Cullins, were characterized biophysically [50].

The ASB proteins also belong to the class of SOCS-box proteins. The ASB subclass in humans consists of 18 members that feature a C-terminal SOCS-box and a variable number of ankyrin repeats at the N-terminus. The ankyrin repeat domain serves as a PPI module to recruit the substrate protein. The SOCS-box domain of the ASB family members recruits Cul5 via the EloBC adaptor complex to form an active E3 ubiquitin ligase complex. Crystallographic and biophysical studies have been conducted recently with one component of the ASB subclass, ASB9, a protein of poorly characterized function but with a potential link to disease [51,52]. These studies have revealed an important structural basis for the Cul5 interaction specificity and for the quaternary ASB9–EloBC–Cul5_NTD_ protein complex assembly [51,52].

VHL binds with EloBC and Cul2–Rbx1 to form CRL2^VHL^, an E3 ligase that targets HIF-1α (hypoxia-inducible factor 1α) for proteasomal degradation [53,54]. HIF-1α is a transcription factor that plays a crucial role in oxygen homoeostasis and tumour angiogenesis [55,56]. The key structural determinant for recognition of HIF-1α by VHL is a hydroxyproline residue of the substrate that is hydroxylated under normoxic conditions in an oxygen-dependent manner [57]. As discovered by mass spectrometric analysis, there are a number of receptor proteins that form CRL2 complexes in addition to VHL, e.g. LRR-1 (leucine-rich repeat protein 1) and FEM-1 proteins [21,48].

### CRL3

**Figure 3:**
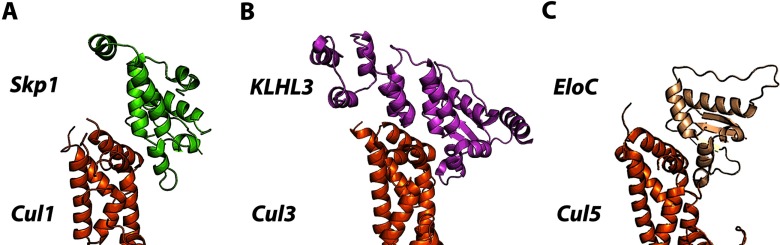
Assembly between adaptor subunits and Cullins (**A**) Skp1–Cul1 (PDB code 1LDK). (**B**) BTB protein KLHL3–Cul3 (PDB code 4HXI). (**C**) ElonginC–Cul5 (PDB code 4JGH). The adaptor proteins bind the N-terminal surface of their respective Cullins to form extended and predominantly hydrophobic interfaces.

The general CRL architecture varies depending on the family member. Remarkably, CRL3 utilizes substrate-specific adaptors that implement a dual adaptor/receptor function within a single polypeptide chain [22,58–60]. These proteins usually contain several domains and are characterized by a common structural motif called the BTB fold that binds the N-terminal end of the Cul3 scaffold. The human genome encodes over 200 BTB proteins [22] that were originally discovered in the bric-a-brac, tramtrack and broad complex transcription factors of *Drosophila melanogaster* [61]. Importantly, comparison with other CRLs shows that BTB–Cul3, ElonginC–Cul2/5 and Skp1–Cul1 interfaces are structurally analogous to each other, although the interacting subunits are not interchangeable [59] (Figure 3).

The functional domains of BTB proteins are fused into an individual subunit; the BTB domain associates with Cul3–Rbx1, whereas the receptor domains, such as the MATH (meprin and TRAF homology, where TRAF is tumour necrosis factor receptor-associated factor) motif, Kelch β-propeller repeat and zinc fingers serve as substrate-recognition elements [23]. Interestingly, CRL3 complexes can dimerize via their BTB domains to acquire two substrate receptors and two catalytic RING domains, as demonstrated for SPOP (speckle-type POZ protein) that contains both BTB and MATH domains [62]. Crystal structures of KLHL3–Cul3_NTD_ (where KLHL is Kelch-like protein) [63] and KLHL11–Cul3_NTD_ [64] have revealed the details of the molecular interface between the BTB proteins and the Cul3 scaffold.

One of the most studied BTB proteins is Keap1 (Kelch-like enoyl-CoA hydratase-associated protein 1). Keap1 contains a Kelch motif for recognition of substrate Nrf2 (nuclear factor-erythroid 2-related factor 2) transcription factor that plays an important role in the oxidative stress response [65]. Structural information on substrate recognition is also available for other Kelch-domain receptors. CRL3 receptor subunits KLHL2 and KLHL3 target for ubiquitination the substrate WNK4 (with no lysine 4) kinase, which plays key roles in blood pressure regulation [66]. The biological function of CRL3s, their substrates and relevant disease implications, as well as the prospects of therapeutic targeting of this subfamily of enzymes, have been reviewed recently [67–69].

### CRL4A AND CRL4B

Scaffold proteins Cul4A and Cul4B share over 80% similarity at the sequence level, with the main difference being that Cul4B contains an extended NTD. Despite being closely related, their functional roles in human disease are very different. Cul4A is mainly involved in oncogenesis, whereas Cul4B was found to play a role in X-linked mental retardation [70]. Despite structural similarities of both Cul4A and Cul4B to other members of the Cullin family, there is a major difference in their architecture compared with other CRLs. The CRL4 assembly employs adaptor DDB1 that is significantly distinct from BTB, Skp1 and ElonginC proteins in terms of its structure, function and larger size. The details of CRL4 assembly were revealed from the crystal structures of DDB2–DDB1–Cul4A–Rbx1, DDB2–DDB1–Cul4B–Rbx1 [71] and a virally hijacked SV5 (simian virus 5) V protein–DDB1–Cul4A–Rbx1 complex [72]. The DDB1 adaptor is made up of three WD40 β-propeller domains (BPA, BPB and BPC) and serves to recognize UV-induced damaged DNA lesions. A pair of coupled BPA and BPC domains recruits the DCAF (DDB1–Cul4A-associated factor) substrate receptor subunit and a third flexibly connected BPB domain associates with the Cul4 scaffold.

The DCAF protein class, also known as DWD (DDB1-binding WD40) and CDW (Cul4–DDB1-associated WD40 repeat), share a conserved structural WD40 β-propeller domain [73]. They were originally found to bind the DDB1–Cul4A complex in a tandem-affinity-assisted proteomics study [72]. Out of over 300 WD40-containing proteins encoded in the human genome, there are nearly 90 DCAFs that could potentially bind the DDB1 adaptor and be incorporated into CRL4 as substrate-recognition subunits [74]. Docking of DCAF receptors and viral proteins to the DDB1 adaptor occurs by means of a specific structural determinant: the short helical H-box motif [75,76]. Another receptor motif, the double-DXR box, plays an important role in the interaction between DCAF and the DDB1 adaptor [72]. Examples of DCAF proteins include DDB2, CSA (Cockayne syndrome A), Cdt2 (Cdc10-dependent transcript 2) and VPRBP (Vpr-binding protein, where Vpr is viral protein R). DCAF proteins are involved in DNA replication and damage repair, cell cycle and tumour suppression [70,73,77]. Interestingly, CRL4^DDB2^ is significantly different from other CRL family members in that it binds DNA; however, the ultimate targets for ubiquitination are the proteins located in close proximity [71]. Structural and biochemical studies revealed that binding of damaged DNA to the DDB2 receptor of the CSN [COP9 (constitutive photomorphogenesis 9) signalosome complex]–CRL4^DDB2^ complex leads to discharge of CSN subunit 1 that is bound at the C-terminal site of the Cullin scaffold (substrate-activated CSN release). The role of CSN as a regulator of CRL activity was originally shown for CRL4^DDB2^ and CRL4^CSA^ ligases in studies using UV-induced DNA damage [78]. Surprisingly, F-box proteins β-TrCP and Fbw5 were reported as substrate receptors for CRL4^β-TrCP^ [79] and CRL4^Fbw5^ [80] targeting inhibitors of mTOR signalling REDD1 (regulated in development and DNA damage response 1) and TSC2 (tuberous sclerosis protein 2) respectively. However, the structural basis of the interaction between these particular F-box proteins and the DDB1 adaptor has so far remained elusive.

### CRL7

Cul7 was initially identified as a member of the Cullin family in mass spectrometric and immunoprecipitation studies showing that it forms a Fbxw8–Skp1–Cul7–Rbx1 E3 ubiquitin ligase complex [81,82]. Although Cul7 comprises a Cullin homology domain like other members of the family, it stands aside because of its distinctive structural characteristics such as the significantly larger size and the presence of Doc (also present in the APC-associated protein Doc1/APC10) and CPH [conserved within Cul7, PARC and HERC2, where HERC2 is HECT domain- and RLD (regulator of chromosome condensation 1 protein-like domain) domain-containing E3 ubiquitin protein ligase 2] domains. The Doc domain of Cul7 is similar to the one in APC10 and HERC2 [83]. The CPH domain that is also conserved in PARC and, interestingly, HERC2 was found to bind the tetrameric form of p53 [84]. Overall, the multisubunit assembly of CRL7 is very similar to CRL1 in that it involves the Skp1 adaptor, an F-box protein receptor and Rbx1 [81]. However, unlike Cul1, Cul7 does not directly bind Skp1 alone, but only the Skp1–Fbxw8 complex.

Fbxw8 contains two functional domains (F-box and WD40 repeat) and it is the only F-box protein identified as a substrate receptor subunit of CRL7 so far [85]. Interestingly, Fbxw8 was discovered to mediate heterodimerization of CRL1 and CRL7 forming a high-order ubiquitin ligase complex that plays a role in placental development [86]. Both RING subunits of the resulting Rbx1–Cul1–Skp1–Fbxw8–Cul7–Rbx1 complex could potentially be involved in the interaction with E2 and substrate ubiquitination. Further immunoprecipitation studies revealed that mutants of Fbxw8 lacking the F-box domain retained the ability to bind Cul7, but not Cul1. In contrast, it was shown that WD40-deficient mutants did not bind Cul7, but were still able to interact with Cul1. Finally, mutants lacking the linker region between F-box and WD40 domains could not bind either Cul1 or Cul7. Overall, on the basis of these results, it was proposed that Cul7–Rbx1 interacts with the WD40 domain region of Fbxw8 and Skp1–Cul–Rbx1 binds the F-box domain [86]. Therefore, according to this model, it appears that, in principle, Cul7 does not form any interaction with the Skp1 adaptor and instead directly recruits the substrate receptor Fbxw8. However, detailed structural information on the exact mode of the assembly has yet to be obtained.

A few reported substrates of CRL7^Fbxw8^ include IRS-1 (insulin receptor substrate 1) [87] that mediates the insulin signalling processes, cyclin D1 that is involved in cell cycle progression [88] and also the protein Grasp65 (Golgi reassembly stacking protein 65) that plays a role in regulation of neuronal Golgi morphogenesis and dendrite elaboration [89]. It was also demonstrated that mutations in the *Cul7* gene result in a number of hereditary human diseases related to growth retardation [90,91].

## CRL REGULATION

In many cases, normal functioning of CRLs under physiological conditions depends on the interplay between regulatory proteins such as NEDD8, CAND1 (Cullin-associated NEDD8-dissociated protein 1) and CSN. Together, these proteins alter the activity of CRLs and modulate the association/dissociation cycles of CRL subunits [92].

### NEDD8

NEDD8 is a ~9 kDa protein that shares over 50% sequence identity with ubiquitin. NEDD8 is covalently conjugated to a specific conserved lysine residue at the Cullin CTD in a process termed NEDDylation [93,94]. Similarly to ubiquitin, the NEDD8 modification pathway involves its own set of dedicated E1 and E2 enzymes. NEDDylation was initially reported for Cul1 in yeast [95,96]. NEDD8 modification is known to regulate CRL activity and mediate ubiquitination of substrates. For example, NEDD8 was demonstrated to be essential for ubiquitination of p27 by CRL1 [97] and also IκBα and β-catenin by CRL1^β-TrCP^ [98]. NEDD8 modification was also reported to promote the binding of E2~Ub conjugates to CRL [99].

The crystal structures and SAXS data of Cul5_CTD_–Rbx1 and NEDD8~Cul5_CTD_–Rbx1 complexes shed crucial light on the potential mechanism of action [100]. This study revealed that Cullin NEDDylation reorients the modified Cullin subdomain and dramatically alters the structural flexibility of the Rbx1 RING domain. In this open form, Rbx1 moves away from its interaction with the Cullin and partly closes the gap between the E2 and the substrate. Further biochemical data support the hypothesis that this conformational change stimulates ubiquitin transfer.

NEDD8-mediated regulation of CRL activity was demonstrated for all members of the Cullin family, except for Cul7 [101,102]. Remarkably, on the basis of immunoprecipitation studies, Cul7 was reported not to be NEDDylated in cells, but the closely related PARC (sometimes called Cul9) still undergoes NEDD8 modification [103]. The role of the NEDD8 conjugation pathway in cancer has been reviewed extensively [104,105].

### CAND1

CAND1 is a ~136 kDa regulatory protein that reversibly binds to unNEDDylated Cullin scaffold and modulates the CRL assembly by competing with the adaptor subunit. This regulatory mechanism was first shown for CRL1 [106–108]. NEDDylated CRLs do not recruit CAND1. Conversely, CAND1 inhibits NEDDylation by restricting access of NEDD8 to the conserved acceptor lysine residue of Cullin [109]. CAND1 was reported to substantially inhibit ubiquitination of substrate IκBα by CRL1^β-TrCP^ by interfering with the adaptor assembly [110]. The same study showed CAND1 can interact with Cul1, Cul2, Cul3, Cul4A and Cul4B. Later it was established that CRL1, CRL4B and CRL5 have a significant level of interaction with CAND1; however, other CRLs show only relatively low levels of CAND1 association [111]. Crystal structures of CAND1–Cul1–Rbx1 [112] and CAND1–Cul4B–Rbx1 [71] revealed that CAND1 binds by wrapping its ends around both the NTD and the CTD of the Cullin scaffold.

Recently, it was demonstrated that CAND1 functions as an exchange factor for the adaptor–receptor complex in CRL1 [113–115]. According to the proposed models, CAND1 promotes the dissociation of an incorporated adaptor–receptor complex and, as a result, facilitates recruitment of another partner instead. This process is postulated to determine the variable repertoire of incorporated substrate receptors and thus to modulate the functional activities of CRLs. Studies using FRET-based assays demonstrated that CAND1 significantly enhances dissociation of the adaptor–receptor complex from the Cullin scaffold [114]. It was proposed that substrate binding stimulates NEDDylation, which then blocks CAND1 association resulting in overall CRL stabilization. However, once the substrate is ubiquitinated and released for degradation, then subsequent deNEDDylation of CRL is followed by CAND1 association that enables exchange of the adaptor subunits. Another research group used quantitative MS to show that CAND1 regulates the *in vivo* dynamics of CRL assembly via exchange of F-box substrate receptors [115]. Ultimately, an interesting hypothesis was proposed that the main role of NEDD8 might not be in activating CRLs, but rather in regulating the dynamics of substrate receptor exchange [113].

### CSN

NEDD8 modification is a reversible process and deNEDDylation is conducted by an eight-subunit CSN complex that is another CRL regulatory protein [116]. CSN-mediated cleavage of the isopeptidic bond between NEDD8 and Cullin is carried out by its CSN5 subunit containing the catalytic JAMM (Jab1/MPN/Mov34 domain metalloenzyme) motif acting as a zinc metalloprotease active site [117,118]. Recently, the crystal structure of CSN revealed the multisubunit organization of the protein complex [119]. The functionally important cycles of NEDDylation/deNEDDylation of CRLs regulated by the CSN have implications in cancer [120].

## CRL OLIGOMERIZATION

In some cases, CRL activation and functioning requires oligomerization of the E3 complex. The biological implications of CRL oligomerization as found to date include activity regulation, enhancement of substrate ubiquitination and mechanistic aspects of ubiquitin transfer.

Oligomerization can occur via the adaptor, receptor or Cullin scaffold, or their combinations. Examples include BTB proteins such as the homodimeric adaptor SPOP in complex with Cul3_NTD_ and heterodimeric SPOP–SPOPL (SPOP-like) complex that regulates CRL activity by determining its oligomeric state [121]. A similar example describes high-order oligomerization of SPOP that promotes substrate ubiquitination by CRL3^SPOP^ [122]. Homodimerization of another BTB protein MEL26 (maternal effect lethal 26) in CRL3^MEL26^ was reported to be important for its E3 ligase activity [60]. Another BTB protein, KLHL3, in complex with Cul3_NTD_ was shown to homodimerize via its BTB–BACK (BTB and C-terminal Kelch) domain [63]. A two-site model for substrate recognition was proposed for CRL3^KLHL11^ based on the crystal structure of KLHL11–Cul3_NTD_ [64]. The F-box substrate receptors β-TrCP1 and β-TrCP2 can form homo- and hetero-dimers via their the N-terminal regions [123]. The homodimers CRL1^β-TrCP1−β-TrCP1^ and CRL1^β-TrCP2−β-TrCP2^ selectively target substrate IκBα for proteasomal degradation. In addition, yeast F-box proteins Pop1p and Pop2p can homo- and hetero-oligomerize to regulate diverse functional activity of the E3 ligase [124,125]. Substrate receptor subunit DCAF1 can also dimerize to enhance the activity of CRL4A^DCAF1^ [126].

## VIRAL HIJACKING OF CRLs

CRLs or their components can be hijacked by viral proteins to manipulate the host cellular processes, and to exploit the host UPS in order to promote viral replication [127,128]. Examples of CRL hijacking have been reported for several viral proteins. HIV-1 Vpu (viral protein U) protein binds the β-TrCP receptor of CRL1 to target T-cell surface glycoprotein CD4 for proteasomal degradation [129]. HIV-1 Vpr was found to associate with CRL4A^VPRBP^ and to trigger cell cycle arrest [130,131]. SV40 large T-antigen was found to bind F-box receptor Fbw7 of CRL1 and regulate the turnover of substrate cyclin E [132]. HPV (high-risk human papillomavirus) E7 was shown to interact with the receptor Skp2 and be targeted for ubiquitination by CRL1^Skp2^ [133]. Adenovirus proteins E4orf6 and E1B55K were shown to hijack the EloBC–Cul5–Rbx1 complex and promote ubiquitination of tumour-suppressor protein p53 [134].

Several types of paramyxoviruses, including HPIV-2 (human parainfluenza virus type 2) and SV5 are able to hijack the CRL4A machinery [135]. These viruses produce a conserved V protein that recruits the DDB1 adaptor of CRL4A and promotes ubiquitination of substrate STAT1 and its targeted proteasomal degradation [136,137]. The crystal structure of DDB1 in complex with V protein revealed the structural basis of the interaction [138].

HIV-1 Vif (virion infectivity factor) protein suppresses the host antiviral activity by hijacking the CRL5 machinery. Specifically, Vif recruits the EloBC–Cul5–Rbx1 complex to form CRL5^Vif^ and induce proteasomal degradation of the substrate APOBEC3G (apolipoprotein B mRNA-editing enzyme, catalytic polypeptide-like 3G), a host protein that functions as an antiviral factor by inhibiting retrovirus replication [139]. The recently solved crystal structure of Vif–CBFβ–Cul5–EloBC (CBFβ is core binding factor β) uncovered the details of the hijacking mechanism [140]. The mechanism of viral hijacking was shown to involve extended interaction with cellular transcription factor CBFβ that promotes Vif-mediated degradation [141].

The area of viral CRL hijacking becomes more attractive for drug discovery as the number of solved crystal structures increases. Small-molecule inhibitors disrupting interaction of viral proteins with the CRL machinery or blocking the activity of the hijacked E3 ligase complex could lead to the development of novel antiviral therapies.

## DRUG DISCOVERY AND DEVELOPMENT STRATEGIES AND EFFORTS TO TARGET CRLs

Drug discovery in the UPS has seen significant progress in the last decade with the first-marketed proteasome inhibitors bortezomib (Velcade®, Millennium Pharmaceuticals) [163] and more recently carfilzomib (Kyprolis®, Onyx Pharmaceuticals) [164] being approved by the U.S. FDA (Food and Drug Administration) for treatment of multiple myeloma/mantle cell lymphoma. Although proteasome inhibitors are reasonably selective against cancer cells to induce apoptosis, they suppress proteasome-mediated degradation of many proteins in the cell, leading to high risk of toxic side effects. In addition, clinical evidence for growing resistance against proteasome inhibitors is beginning to emerge [165]. Therefore a narrower and more selective targeting of the ubiquitination cascade upstream of the proteasome is being widely accepted as a promising therapeutic approach that may alleviate some of the limitations associated with proteasome inhibitors. The E3 ubiquitin ligases, including the CRLs, capture particular attention in this regard as they are responsible for the specificity of substrate ubiquitination. On the other hand, one must also consider that targeting proteasome activity could benefit from target/pathway promiscuity and multiple mechanisms of action, whereas blocking a single E3 (or a subgroup of E3s) could be functionally overcome via compensatory cellular pathways.

Targeting E3 ubiquitin ligases for drug discovery requires perturbing their PPIs. PPIs are among the most promising targets for therapeutic intervention [166,167], because of the exquisite specificity of many PPIs and their important roles in determining biological function. The use of small organic molecules to modulate PPIs holds many advantages, because of the greater bioavailability of small molecules compared with nucleic acids, peptides or protein therapeutics, and the possibility of oral delivery. However, targeting PPIs using small molecules has traditionally been viewed as challenging because of the perceived difficulty of gaining binding affinity from the large, often featureless, binding interfaces [166,167]. This led to the notion of E3 ligases as undruggable targets. However, targeting E3 ligases with small molecules has been rewarded with some success, particularly against PPIs such as IAP (inhibitor of apoptosis protein)/caspase [168], MDM2 (murine double minute 2)/p53 [169,170], and VHL/HIF-1α [145–148]. These examples give important precedent for the approach and have reinvigorated new efforts against E3 as targets.

It has been proposed that E3 ligases, and CRLs in particular, could be the ‘new’ kinases owing to the substantial therapeutic and market potential of inhibitors for this class of proteins [171]. Importantly, there are a number of issues to consider: (i) unlike kinases, most E3 ligases do not possess a defined ligand-binding site, therefore developing small-molecule inhibitors could be challenging; (ii) most E3 ligases consist of multiple independent subunits that work together in a complex, which means that there could be a number of sites for potential chemical intervention; and (iii) there is no general approach for targeting E3 ligases and each protein will have to be addressed individually according to its specific structural and functional characteristics.

There is currently a growing interest in developing small molecules against CRLs for clinical application, and this area of research could become one of the most attractive for drug discovery in the coming years. Targeting PPIs within CRLs poses specific challenges, owing to the lack of a classical catalytic active site and the large number of potential CRL substrates that could give rise to specificity problems. On the other hand, the complexity of the target system provides opportunities too. For example, the diversity of protein interfaces and surfaces that are accessible within the CRL multi-component complexes provide many potential pockets for small molecules to bind to. This could result in different biological effects and functional outcomes. Small molecules could act directly by inhibiting receptor–substrate interaction, disrupting CRL assembly, as well as allosterically inducing conformational changes leading to suppressed activity or altered ensemble dynamics [172]. In some cases, potent ligands could exert their functional effect through stabilization of certain PPIs within the complex, rather than disruption [173]. Ultimately, this diversity increases opportunities to identify multiple chemical series with different mechanisms of action, thereby maximizing chances of success for drug discovery. An alternative way to prevent CRL–substrate interaction is to act upstream by blocking post-translational modification (i.e. phosphorylation) of the substrate.

Interestingly, compounds that bind and recruit CRLs with high affinity can have unusual, but highly promising, applications as proteolysis-targeting chimaeric molecules (Protacs). Protacs are a class of hetero-bivalent small molecules that promote ubiquitination and subsequent degradation of a target protein of interest by simultaneously binding to and bringing together both the E3 ligase and the target [174,175]. The most attractive feature of Protacs as a potential therapeutic strategy is the ability to remove the target protein from the cell, in addition to, or instead of, inhibiting it.

Strategies for targeting CRLs that have been described in the literature include: (i) *in vitro* screening using functional assays, e.g. ubiquitination assays [176]; (ii) the use of computer programs, e.g. ICM-Pocket Finder, for predicting potential druggable pockets, including those at protein–protein interfaces, and subsequent docking-based virtual ligand screening *in silico* [177]; and (iii) rational structure-based and fragment-based design [145,147]. Each approach has its own advantages, but also drawbacks and limitations; for example, functional screening does not provide direct evidence regarding the binding site of the hits; computational methods for pocket prediction have limited reliability and also require structural information, ideally of bound complexes, to be available; fragment-based approaches strongly rely on numerous biophysical techniques, require often laborious structural optimization and tend to be limited by the low ligand efficiency associated with small molecules targeting PPIs [178].

In our opinion, structure-based drug design is one of the most effective strategies for developing specific small molecules targeting CRLs. Availability of structural data is the main limitation of this approach given that crystallization of large multisubunit protein complexes, especially full-size CRLs, can be notoriously difficult. In addition, NMR spectroscopic studies can be challenging due to the large size of the full complexes and their multisubunit composition [179]. We performed a thorough search within the PDB for crystal structures of CRLs, their individual components and other related proteins published to date (data summarized in Supplementary Table S1). This table could be useful for new structure-based initiatives aiming at the rational design of potent CRL inhibitors/modulators. Most of the solved structures represent individual components or their complexes for CRL1, CRL3, CRL4A/B and CRL5, with only a few examples for CRL2 and no crystal structures for CRL7. The vast majority of these structures provide structural information on substrate–receptor, receptor–adaptor and adaptor–Cullin interfaces. As described in the cases below, some of these interfaces are already being targeted for potential therapeutic intervention. There are only two examples of crystal structures containing substrate receptor, adaptor, Cullin and Rbx subunits altogether: Skp2–Skp1–Cul1–Rbx1 [29] and DDB1–DDB2–Cul4A–Rbx1 [71]. Structures containing full-length Cullin scaffold are only available so far for Cul1–Rbx1 [29], DDB1–Cul4A–Rbx1 and Cul4B–Rbx1 [71,72].

The E3–E2 interplay is responsible for ubiquitin transfer and therefore is also considered to be attractive for small-molecule modulation. In general, this interaction is rather weak and labile, with *K*_D_ values in the low micromolar range [24], therefore even moderately potent, yet selective, ligands could act as effective modulators of this PPI. To date, there are only a few crystal structures of E3–E2 complexes available; however, most of them are non-CRL E3 ligases, as reviewed in [24,180], and will not be covered here. To the best of our knowledge, the only exception is the recently reported structure of the Rbx1–Ubc12~NEDD8–Cul1–Dcn1 (where Ubc12 is ubiquitin-conjugating enzyme 12 and Dcn1 is defective in Cullin NEDDylation 1) complex [181]. The authors provided a structural characterization of an intermediate NEDDylated complex that aids the elucidation of the mechanism of NEDD8 ligation to the Cullin scaffold and substrate ubiquitination. Importantly, the overall architecture of the E3 machinery and its catalytic activity were found to be substrate-regulated. The study represents a major step forward in understanding the mechanistic details of substrate ubiquitination via RING E3–E2~UBL (UBL is ubiquitin-like protein) complexes that had previously remained elusive.

**Figure 4:**
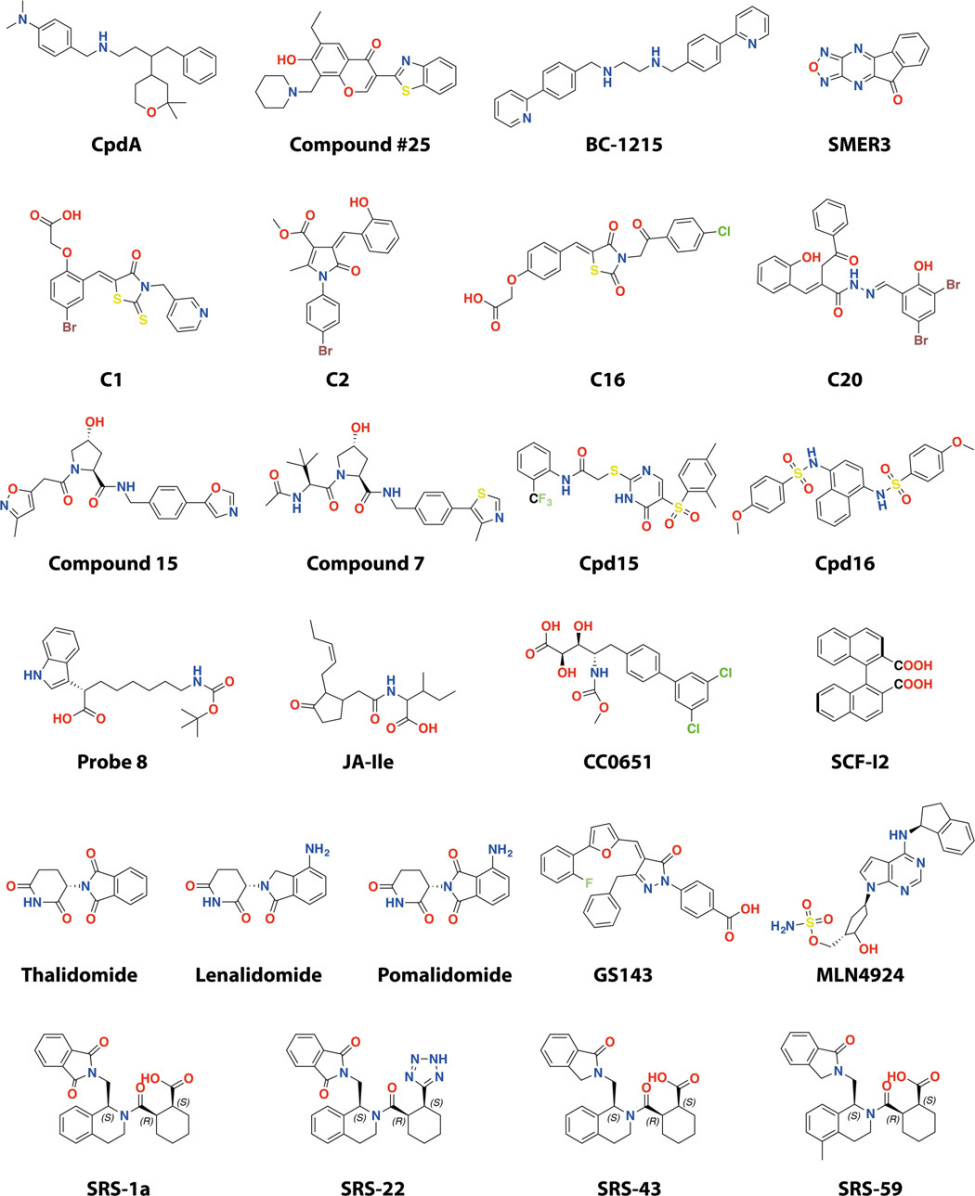
Chemical structures of published small-molecule modulators of CRL activity

The small-molecule ligands of CRLs reported target different components of the CRL complexes; however, in most cases, these act as inhibitors by disrupting the substrate–receptor interface (Figure 4 and Table 2). Several examples are covered in detail in the next section.

**Table 2:** Reported CRL inhibitors, their targeted subunits, mechanism of action and available X-ray crystal structures N/A, not available.

Molecule ID	Target	Function/mechanism	*K*_D_ or IC_50_	PDB code	Reference(s)
CpdA	Skp2 of CRL1^Skp2^	Prevents Skp2 incorporation into CRL1^Skp2^ E3 ligase, possibly by disrupting Skp1–Skp2 interaction	IC_50_=4.2–13.2 μM (against several cell lines)	N/A	[142]
Compound #25	Skp2 of CRL1^Skp2^	Inhibitor of Skp1–Skp2 interaction	N/A	N/A	[143]
C1, C2, C16, C20	Skp2–Cks1 of CRL1^Skp2^	Inhibitors of PPI between Skp1–Cks1 and substrate p27	N/A	N/A	[144]
Compound 15, Compound 7	VHL of CRL2^VHL^	Disruptors of the interaction between VHL and substrate HIF-1α	*K*_D_=5 μM (Compound 15), 185 nM (Compound 7)	3ZRC, 4W9H	[145–148]
BC-1215	Fbxo3 of CRL1^Fbxo3^	Inhibitor of substrate Fbxl2 ubiquitination	IC_50_=0.9 μg/ml	N/A	[149]
SMER3	Met30	Binds to the Met30 receptor of CRL1^Met30^ and inhibits ubiquitination of substrate Met4	N/A	N/A	[150]
GS143	β-TrCP1 of CRL1^β−TrCP1^	Inhibitor of substrate IκBα ubiquitination	IC_50_=5.2 μM	N/A	[151]
Probe 8	TIR1 of CRL1^TIR1^	Disruptor of interaction between TIR1 of CRL1^TIR1^ and substrate Aux/IAA	N/A	3C6N	[152]
JA-Ile	COI1 of CRL1^COI1^	Promotes interaction between COI1 and substrate JAZ1	N/A	N/A	[153]
Thalidomide, lenalidomide, pomalidomide	CRBN of CRL4A^CRBN^	Modulators of CRL4A activity via binding to CRBN subunit	*K*_D_=8.5 nM (thalidomide) (by SPR [154]); 250 nM (thalidomide), 178 nM (lenalidomide), 157 nM (pomalidomide) (measured by FP [155])	4CI1, 4CI2, 4CI3, 4TZ4, 4TZU, 4TZC, 3WX2	[154–156]
CC0651	Cdc34	Inhibitor of E2-conjugating enzyme Cdc34	IC_50_=1.72 μM (inhibition of p27^Kip1^ ubiquitination)	3RZ3	[157,158]
	Cdc34A~Ub	Stabilizes Cdc34A~Ub thioester link	IC_50_=18 μM (inhibition of β-catenin ubiquitination)	4MDK	
SCF-I2	Cdc4	Allosteric inhibitor of Cdc4 binding of phosphorylated substrates	IC_50_=6.2 μM	3MKS	[159]
MLN4924	NAE	Forms a covalent adduct with NEDD8 and inhibits NAE	IC_50_=4.7 nM	3GZN	[19]
SRS-1a, SRS-22, SRS-43, SRS-59, Cpd15, Cpd16	Keap1 of CRL3^Keap1^	Inhibitors of the interaction between Keap1 and substrate Nrf2	IC_50_=2.3 μM (SRS-1a), 7.4 μM (SRS-22), 1.1 μM (SRS-43), 0.75 μM (SRS-59), 118 μM (Cpd15), 2.7 μM (Cpd16)	4L7B, 4L7C, 4L7D, 4N1B, 4IN4, 4IQK	[160–162]

## TARGETING CRLs

### CRL1^Skp2^

The growing evidence in support of the correlation between CRL and cancer or other diseases leads to increased efforts to develop potent inhibitors. The accumulated biological data for CRL1^Skp2^ lays out a solid rationale for stabilization of the substrate p27, i.e. by targeting the p27–Skp2 PPI or other interfaces within the CRL1^Skp2^. Research in this area has already successfully employed virtual screening and other computer-aided methods. Given the relatively large number of published crystal structures of CRL1^Skp2^ components individually or in complex with other proteins (Supplementary Table S1), we propose that a structure-based drug design approach would be particularly attractive for future development of potent inhibitors.

A set of compounds has been developed starting from a virtual screening *in silico* for inhibitors of Skp2-mediated p27 degradation [144]. The authors applied the ICM PocketFinder (Molsoft) program on the published crystal structure [33] to examine the interface formed by receptor Skp2, substrate p27 and the accessory protein Cks1 that is required for the interaction. The approach is based on calculating volume/area ratios to predict druggable surface pockets and was originally described for F-box protein β-TrCP [177]. The pocket identified at the Skp2–Cks1–p27 interface was then targeted by virtual screening of 315000 compounds and followed up by the assay validation. Four hits, C1, C2, C16 and C20, were confirmed to stabilize p27 and inhibit its ubiquitination in a Skp2-dependent manner. These small molecules are postulated to bind specifically at the Skp2–Cks1 interface and disrupt the interaction with p27. Later it was established that C2 and C20 increase the nuclear levels of p27 and inhibit cancer cell proliferation [182], suggesting that these compounds could be further optimized into potent inhibitors to treat CRL1^Skp2^-dependent cancers. Altogether, these proof-of-principle results confirm that the CRL–substrate interface can be successfully targeted with small molecules.

A high-throughput screening was used in the format of an *in vitro* ubiquitination assay of CRL1^Skp2^ to identify the small molecule CpdA (compound A) [142]. The authors propose that the compound acts by disrupting the Skp1–Skp2 interface and preventing Skp2 from incorporating into CRL1^Skp2^ as a substrate receptor domain. CpdA specifically inhibits ubiquitination and induces stabilization of p27^Kip1^, as well as several other substrate proteins (p21^Cip1^ and p57^Kip2^). Interestingly, when CpdA was tested in combination with bortezomib, the activity of each compound was enhanced synergistically. However, CpdA demonstrated relatively low potency in cells that might restrict its applicability *in vivo*.

Another research group applied a high-throughput *in silico* screening strategy to search for compounds that bind to Skp2 and disrupt its interaction with Skp1 [143]. Initially, a library of 120000 compounds was screened to identify 25 potential Skp2–Skp1 interaction inhibitors. One molecule called Compound #25 was firmly validated in an *in vitro* pull-down assay and dose-dependent binding experiments. Compound #25 showed selective binding to Skp2 and did not affect other adaptor F-box proteins (e.g. Fbw7 or β-TrCP) that also interact with Cul1. Although Compound #25 prevented the formation of the Skp2–Skp1 complex and inhibited CRL1^Skp2^ activity, it could not disrupt a pre-existing complex *in vitro*. The authors also demonstrated that Compound #25 inhibited p27 ubiquitination by CRL1^Skp2^, enhanced p27 levels and exhibited tumour-suppressing activity in animal studies.

Interestingly, direct targeting with small molecules is not the only way to modulate CRL activity. Two compounds, SMIP001 and SMIP004 (small-molecule inhibitor of p27 depletion 001 and 004), were found to down-regulate Skp2 by lowering its mRNA levels and negatively affect CRL1^Skp2^ activity [183].

### CRL1^Fbxo3^

F-box proteins are responsible for substrate recognition and therefore represent very attractive drug targets, particularly at the receptor–substrate interface. Recently, the benzathine derivative BC-1215 was developed as an inhibitor of Fbxo3 protein that blocked CRL1^Fbxo3^-mediated ubiquitination of substrate Fbxl2 [149,184]. The benzathine scaffold was identified from molecular docking of 6507 approved/experimental drugs against Fbxo3. BC-1215 disrupted the Fbxo3–Fbxl2 interaction and showed anti-inflammatory properties in a mouse model of cytokine-driven inflammation.

### CRL1^Cdc4^

**Figure 5:**
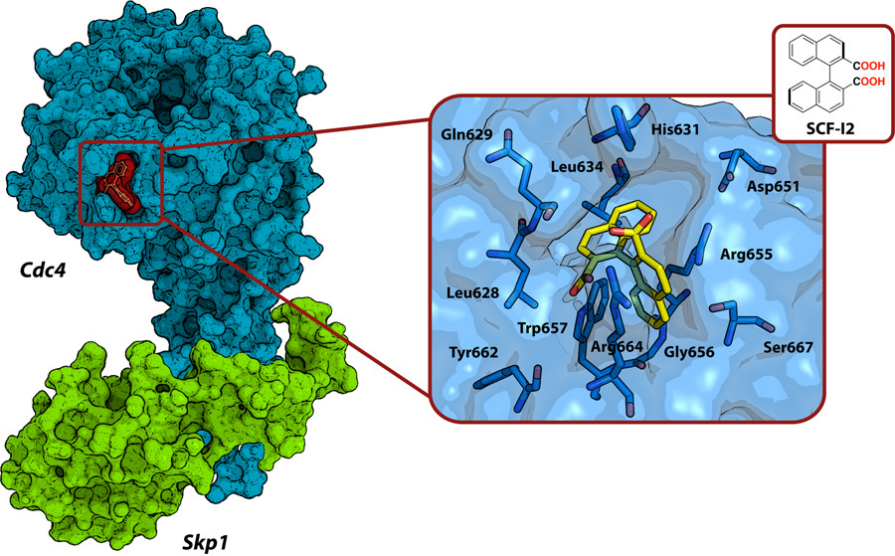
Crystal structure of Skp1–Cdc4–SCF-I2 complex (PDB code 3MKS) Left: the Cdc4–Skp1 protein complex and bound SCF-I2 ligand are shown as molecular surface representations. Right: the expanded inset shows the key residues of Cdc4 (light blue carbons) forming the protein interface that binds SCF-I2 and the ligand chemical structure (yellow carbons); oxygen atoms are in red, and nitrogen atoms are in blue.

Although many of the CRL inhibitors directly target the substrate–receptor interface, there is an interesting example of small molecule SCF-I2 that exerts long-range allosteric modulation of the yeast F-box protein Cdc4 to disrupt its interaction with substrate Sic1 and prevent subsequent ubiquitination of Sic1 by CRL1^Cdc4^ [159] (Figure 5). The biplanar dicarboxylic acid ligand SCF-I2 was discovered by screening a library of ~50000 compounds using an FP (fluorescence polarization) assay. The compound constitutes a racemic mixture; however, only the (*R*)-(+) enantiomer was found to bind Cdc4. Structural studies revealed that SCF-I2 intercalates between adjacent blades 5 and 6 of the conserved WD40 β-propeller domain of Cdc4 in such a way that it induces formation of its own binding pocket located at ~25 Å (1 Å=0.1 nm) distance from the substrate-binding site. Interestingly, this pocket is not present in the apo-form of the Skp1–Cdc4 complex [185]. SCF-I2 was selective towards Cdc4 and showed only little effect on related F-box proteins including the human orthologue of Cdc4 (Fbw7) and the yeast homologous protein Met30. Although SCF-I2 demonstrated significant activity *in vitro*, it failed to inhibit Cdc4 *in vivo* probably because of its poor cell permeability. The authors propose that similar allosteric approaches could be employed for developing inhibitors of other WD40 domain proteins that serve as receptor subunits within many CRLs.

### CRL1^Met30^

Specific disruption of receptor–adaptor interfaces could be an attractive alternative approach to targeting receptor–substrate interactions. A selective CRL1^Met30^ inhibitor called SMER3 (small-molecule enhancer of rapamycin 3) was discovered in a phenotype-based screen of an ~30000 compound library in yeast [150]. Further studies revealed that SMER3 functions by binding to the Met30 substrate-recognition subunit and disrupting its interaction with adaptor Skp1, therefore inhibiting the activity of CRL1^Met30^ and preventing the ubiquitination of substrate Met4. The inhibitor demonstrated high selectivity for Met30 when tested against several other F-box proteins, including Cdc4 and Fbw7.

### CRL1^β-TrCP^

Some NF-κB-regulated inflammatory and cancer processes are mediated by CRL1^β-TrCP^ through ubiquitination and degradation of IκBα, a suppressor of NF-κB nuclear translocation. GS143, a small-molecule inhibitor of CRL1^β-TrCP^, was identified in a FRET-based high-throughput screening of ~50000 compounds [151]. The compound selectively prevented ubiquitination of IκBα and demonstrated anti-inflammatory activity in cellular studies. However, no information was provided to confirm direct binding of GS143 to either β-TrCP or IκBα. The authors propose that the compound's mode of action could involve an interaction with both β-TrCP and phosphorylated substrate IκBα.

### CRL1^TIR1^

**Figure 6:**
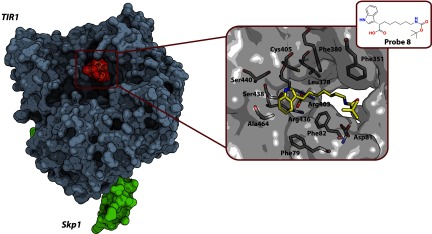
Crystal structure of the Skp1–TIR1–Probe 8 complex (PDB code 3C6N) Left: Skp1–TIR1 protein complex with bound Probe 8 ligand that blocks the interaction of TIR1 with substrate Aux/IAA. Right: the key TIR1 residues (grey carbons) and Probe 8 (yellow carbons) are shown as sticks; oxygen atoms are in red, and nitrogen atoms are in blue.

Auxin is a gene-regulatory plant hormone that is crucial for CRL1^TIR1^-mediated ubiquitination and degradation of substrate Aux (auxin)/IAA (indole-3-acetic acid) transcriptional repressor proteins. Previously, a crystal structure was determined for a Skp1–TIR1 complex (TIR1 is transport inhibitor response 1) alone and with several auxin molecules bound [186]. It was revealed that auxin binds in the substrate-binding pocket of TIR1 and promotes the further recruitment of Aux/IAA. Additionally, a small molecule inositol hexakisphosphate was found bound to TIR1, and presumed to be a cofactor of the auxin receptor. Later, the same group reported the development of small-molecule inhibitors of the F-box protein TIR1 that serves as substrate receptor domain of the CRL1^TIR1^ E3 ligase [152]. Substrate Aux/IAA binds to the leucine-rich repeat domain of TIR1 in an auxin-mediated manner. This suggested that auxin analogues could potentially disrupt the interaction. Towards this aim, a series of alkylated derivatives of a natural auxin, IAA, was developed. One compound, called Probe 8, proved to be the most potent in a pull-down assay and demonstrated a TIR1-dependent auxin-antagonistic response in *Arabidopsis thaliana*. Owing to its long alkyl chain, Probe 8 blocks the interaction between TIR1 and Aux/IAA, as supported by the crystal structure of TIR1 in complex with the small molecule (Figure 6). However, no ubiquitination assay data was provided to confirm inhibitory activity of Probe 8 towards CRL1^TIR1^. Nevertheless, the authors suggest that disrupting the auxin-mediated interaction between TIR1 and substrate Aux/IAA could modulate E3 ligase function and potentially prevent the substrate from ubiquitination.

### CRL1^COI1^

A small-molecule-mediated interaction between a CRL and its substrate is not unique to the F-box protein TIR1. A similar mechanism was also identified for its close homologue COI1 (coronatine-insensitive protein 1) and the jasmonate class of plant signalling molecules [153]. Phenotypical studies in plants and the yeast two-hybrid system were used to establish that a repressor of jasmonate signalling, JAZ1 (jasmonate/ZIM domain protein 1, where ZIM is zinc finger expressed in inflorescence meristem), is the substrate of CRL1^COI1^. The plant hormone jasmonoyl-isoleucine (JA-Ile) was demonstrated to promote the interaction between the F-box protein COI1 and the substrate JAZ1, leading to CRL1^COI1^-dependent ubiquitination and proteasomal degradation of JAZ1. Although no inhibitors of the COI1–JAZ1 interaction have been reported to date, it is reasonable to suggest that JA-Ile could be used as a starting point for the development of such compounds, in a manner resembling that of auxin analogue Probe 8, as described above.

### CRL2^VHL^

**Figure 7:**
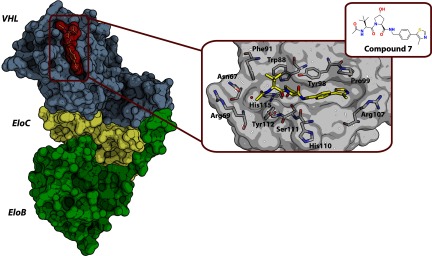
Crystal structure of the VHL–ElonginB–ElonginC–Compound 7 complex (PDB code 4W9H) Left: the protein complex and the ligand are shown as molecular surface representations. Right: the insets show the chemical structure of the ligand and a close-up of its binding site, with protein key residues (grey carbons) and ligand molecule (yellow carbons) shown as sticks; oxygen atoms are in red, nitrogen atoms are in blue, and sulfur atoms are in orange.

HIF-1α is a transcription factor that is targeted for ubiquitination and proteasomal degradation by the VHL E3 ligase CRL2^VHL^. Blocking the activity of VHL would result in up-regulation of HIF-1α which is an attractive therapeutic strategy in certain diseases where triggering the hypoxic response is proven to be beneficial. Our laboratory, in collaboration with scientists from Yale University, developed the first series of inhibitors of the PPI between VHL and HIF-1α [145–147]. VHL serves as a substrate receptor within CRL2^VHL^ and recognizes a key hydroxylated proline residue of HIF-1α [146,147]. The compounds were structurally designed as hydroxyproline derivatives to compete with HIF-1α and then optimized using SARs (structure–activity relationships) based on the crystal structures of the protein–ligand complexes. Compound 15, the best inhibitor until then [*K*_D_=5 μM by ITC (isothermal titration calorimetry) and FP] was then deconstructed and the interaction of its building blocks was studied biophysically. This allowed the dissection of the relative contribution of individual functional groups to the ligand-binding energy and assessment of the feasibility of fragment-based screening for targeting the VHL–HIF-1α interface [145]. More recently, guided by the results of this deconstructive study and by numerous X-ray crystal structures and ITC data, this PPI inhibitor series was optimized further, resulting in Compound 7 (*K*_D_=0.185 μM by ITC) that is currently the most potent VHL/HIF-1α inhibitor reported to date [148] (Figure 7).

### CRL3^Keap1^

**Figure 8:**
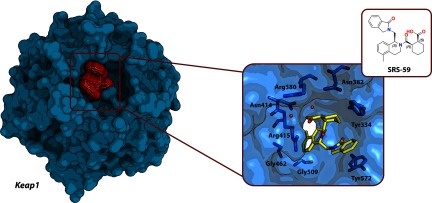
Crystal structure of the Keap1–SRS-59 complex (PDB code 4L7D) Left: Keap1 receptor subunit of CRL3^Keap1^ shown with bound SRS-59 inhibitor that disrupts the interaction with substrate Nrf2. Right: Keap1-binding site with key residues (light blue carbons) and bound SRS-59 molecule (yellow carbons), and the chemical structure of the inhibitor; oxygen atoms are in red; and nitrogen atoms are in blue.

Nrf2 serves as a substrate for CRL3^Keap1^ and plays an important role in inflammatory and cancer pathways [187]. Recently, SRS-1a, a small-molecule inhibitor of the Keap1–Nrf2 interaction was discovered in a high-throughput screening of the NIH's MLPCN (Molecular Libraries Probe Production Centers Network) library [160]. The molecule targets the receptor Kelch domain of BTB protein Keap1. An independent study explored further optimization of SRS-1a, resulting in a highly potent SRS-59, and next established the co-crystal structure of this compound and several of its derivatives in complex with Keap1 [161] (Figure 8). All compounds exhibited low-micromolar potency and good cell permeability, thus providing good starting points for further development. These strategies successfully targeting Keap1 could be also extended to other similar CRL3 targets.

**Figure 9:**
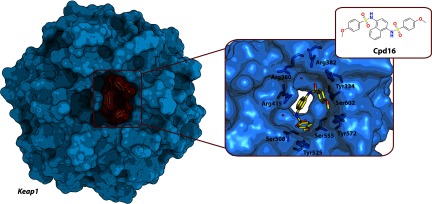
Crystal structure of the Keap1–Cpd16 complex (PDB code 4IQK) Left: structure Keap1 with bound Cpd16 inhibitor of the Keap1–Nrf2 interaction are shown as molecular surface representations. Right: key residues of Keap1 (light blue carbons) and Cpd16 (yellow carbons) are shown; oxygen atoms are in red, nitrogen atoms are in blue, and sulfur atoms are in orange. Cpd16 binds to the same site as the above mentioned SRS-59.

Another example of successful identification of a Keap1–Nrf2 inhibitor involved screening a commercial compound library resulting in the micromolar affinity inhibitors benzenesulfonyl-pyrimidone Cpd15 and benzene-disulfonamide Cpd16 [162] (Figure 9). The latter compound demonstrated up-regulation of Nrf2-response genes in a luciferase cell-reporter assay. X-ray crystal structures were solved for Keap1 in complex with both compounds (PDB codes 4IN4 and 4IQK).

### CRL4A^CRBN^

**Figure 10:**
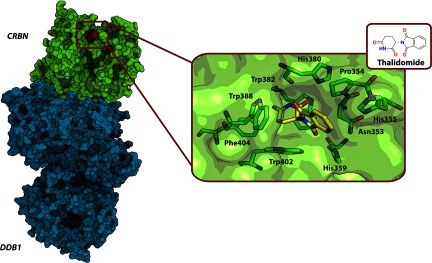
Crystal structure of the CRBN–DDB1–thalidomide complex (PDB code 4CI1) Left: CRBN–DDB1 receptor–adaptor complex of CRL4A^CRBN^ with thalidomide bound to CRBN are shown as molecular surface representations. Right: the residues of CRBN (green carbons) forming the interaction interface with thalidomide (yellow carbons); oxygen atoms are in red, and nitrogen atoms are in blue.

Thalidomide is well known for its teratogenicity in pregnant women; however, nowadays, the drug and its analogues are used for the treatment of multiple myeloma and leprosy. Thalidomide was previously found to exert its teratogenic effect primarily through inhibiting cereblon (CRBN), a protein that interacts with the adaptor DDB1 to form CRL4A^CRBN^ [154]. Later, lenalidomide and pomalidomide, two close analogues of thalidomide, were also found to display their anti-cancer effect also by targeting CRL4A^CRBN^ via direct interaction with CRBN [188,189]. Recently, crystal structures of DDB1–CRBN in complex with thalidomide, lenalidomide and pomalidomide were solved that further verified CRBN as the substrate receptor component of CRL4A^CRBN^ [155] (Figure 10). All three ligands bound in the same pocket of CRBN and demonstrated similar binding modes, and very tight interaction with *K*_D_ values of 250 nM (thalidomide), 178 nM (lenalidomide) and 157 nM (pomalidomide) measured by FP assays. Anti-cancer activity was later found to be due to the compounds binding to CRL4ACRBN and modulating the E3 ligase activity resulting in promoted degradative ubiquitination of several members of the Ikaros transcription factors family in a manner similar to auxin-mediated degradation of Aux/IAA, described above [186]. Using protein microarray analysis, the authors also identified the MEIS2 (myeloid ecotropic viral integration site 1 homologue 2) transcription factor as an endogenous substrate of CRL4A^CRBN^. Independently, the crystal structure was solved for DDB1–CRBN in complex with lenalidomide and an individual TBD (thalidomide-binding domain) of CRBN alone and in complex with thalidomide and pomalidomide [156].

### CRL5^Vif−CBFβ^

Several compounds targeting CRL5^Vif−CBFβ^ were discovered using cell-based and enzyme-based screening assays. Examples include RN-18 [190], MM-1 and MM-2 [191] that reportedly inhibited HIV-1 Vif function; however, the exact structural mechanism of action remains unclear. Recently, the structural basis for hijacking CRL5 was unveiled and Vif was found to incorporate into the E3 ligase by forming a multimeric Vif–CBFβ–EloBC–Cul5 complex [140]. The structural data substantially improves the understanding of viral hijacking and provides further opportunity for rational anti-HIV drug design [192].

## TARGETING UPSTREAM OF CRLs

### Cdc34 E2 enzyme

Targeting substrate–receptor interaction or integral components of the CRL machinery is not the only way to modulate CRL catalytic activity. Alternative approaches include targeting enzymes upstream of the E3, such as E1s and E2s, as well as enzymes responsible for NEDD8 modification of the Cullin scaffold.

**Figure 11:**
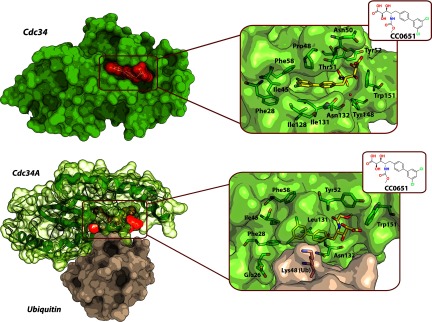
Structures of CC0651 bound to an allosteric pocket on the E2-conjugating enzyme Cdc34 Top: crystal structure of the Cdc34–CC0651 binary complex (PDB code 3RZ3). The protein and its ligand are shown as molecular surface representations. Inset: Cdc34 residues (green carbons) forming the binding pocket and the CC0651 ligand (yellow carbons). Bottom: crystal structure of the ternary complex Cdc34A~Ub–CC0651 (PDB code 4MDK). CC0651 (yellow carbons) is bound embedded within the Cdc34A~Ub covalent conjugate protein. CC0651 suppresses the hydrolysis of the thioester bond between the catalytic cysteine residue of Cdc34 (green carbons, cysteine residue not shown) and ubiquitin (brown carbons, Lys^48^ side chain shown). Oxygen atoms are in red, nitrogen atoms are in blue, and chlorine atoms are in light green.

An interesting example shows how a high-throughput screen for CRL1^Skp2^ inhibitors aided the discovery of an E2 enzyme modulator instead [157]. Here, a screening campaign based on a previously developed ubiquitination assay [142] identified the small molecule CC0651 that inhibited ubiquitination of substrate p27^Kip1^. The compound was not selective towards p27^Kip1^ and demonstrated inhibitory properties against other CRL1-based substrate/receptor pairs; however, biochemical studies established that CC0651 targets the E2-conjugating enzyme Cdc34 operating together with CRL1 [13]. Importantly, the compound showed specificity for Cdc34 compared with other E2 enzymes despite their significant structural similarity. It is well known that the presence of a hydrolysable thioester bond between E2 and ubiquitin is crucial for the catalytic activity of the whole E3 ligase. The crystal structures of Cdc34 and the Cdc34–CC0651 complex revealed that CC0651 targets a previously unknown allosteric pocket at a distance from the catalytic cysteine residue. Ligand binding is observed to induce a significant conformational change in the E2 protein to open up such pocket. In so doing, CC0651 appears to stabilize the E2–ubiquitin interaction and lock the complex in an inactive conformation, therefore inhibiting the discharge of ubiquitin from the E2~Ub conjugate on to the substrate lysine residues (Figure 11). More recently, the same group solved the crystal structure of a Cdc34~Ub–CC0651 complex, and determined that CC0651 bridges the Cdc34~Ub link and suppresses hydrolysis of the weak thioester bond between the two proteins [158]. CC0651 was also found to negatively affect proliferation of cancer cells.

Targeting the intermediate complex CRL–E2~UBL presents attractive opportunities for inhibiting CRL activity. In particular, the canonical hydrophobic interface between the Rbx RING and E2~UBL (e.g. Cdc34~Ub or Ubc12~NEDD8) could be amenable for small-molecule modulation. Potential druggability of the interface can be addressed by targeting the key side-chain interactions that allosterically mediate activation of the E2~UBL conjugate. It was demonstrated recently that the ‘linchpin’ residue Arg^46^ of Rbx1 facilitates RING–E2 interaction for Cdc34~Ub and Ubc12~NEDD8 [181]. Structural studies showed that Arg^46^ brings in close proximity the surface of Ubc12 interacting with the RING domain and the N-terminal loop of NEDD8 in such a way that they both face a distinctive pocket of Rbx1 next to Arg^46^ itself. Importantly, mutation of Arg^46^ prevents NEDD8 transfer from Ubc12 to Cul1. It would therefore seem reasonable that a small molecule targeting this pocket on Rbx1 and disrupting these specific interactions could provide an attractive approach to modulate CRL activity. It is worth pointing out that this interaction is not expected to be significantly affected by CC0651, as this compound targets Cdc34 on a distinct site that is compatible with Rbx1 binding [158].

Another potential target for intervention is the interface formed by the acidic C-terminal tail of Cdc34 and the so-called basic canyon of Cul1 which is close to the Rbx1 interface at the Cul1 CTD [193]. Kleiger et al. [193] used binding assays and *ab initio* docking to demonstrate how rapid dynamics of the high-affinity Cdc34–Cul1 interaction can influence the activity of the CRL E3 ligase. The study describes putative mechanisms of Cdc34 action, including full or partial dissociation from Cul1–Rbx1. It was proposed that the Cdc34 acidic tail could play an important role in mediating the assembly of ubiquitin chains on the substrate. These observations point to the potential of disrupting the interaction between the Cdc34 tail and Cul1 to serve as an attractive drug target site for small-molecule intervention.

### NEDD8–NAE

**Figure 12:**
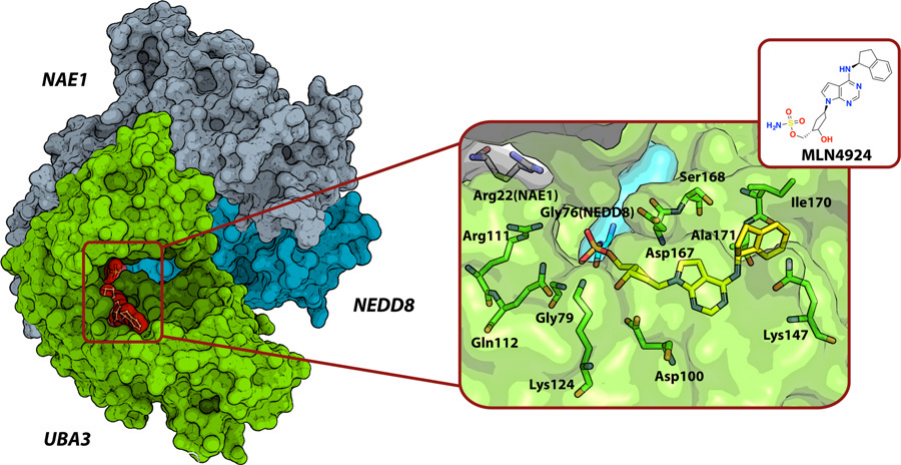
Crystal structure of the NAE1–UBA3–NEDD8~MLN4924 complex (PDB code 3GZN) The NAE1 regulatory subunit and the UBA3 catalytic subunit form the heterodimeric NAE. Left: NAE complexed with NEDD8~MLN4924 covalent adduct, where MLN4924 inhibits the active site of NAE. Right: the residues forming interface between NAE1 (grey carbons), UBA3 (green carbons), NEDD8 (cyan carbons) and MLN4924 (yellow carbons). Oxygen atoms are in red, nitrogen atoms are in blue, and sulfur atoms are in orange.

The ubiquitin-like protein NEDD8 enhances CRL activity by inducing dynamics of the complex and activating conformational shift of Rbx–E2~Ub to bring ubiquitin in close proximity to the substrate bound at the opposite terminus of the E3 ligase [100,194]. CRLs are the main targets for NEDDylation [195], a process of covalent NEDD8 modification of a conserved lysine residue at the Cullin CTD. The conjugation occurs through a pathway involving its own E1 and E2 enzymes. A group of scientists at Millennium Pharmaceuticals developed a highly selective inhibitor of NAE (NEDD8-activating enzyme), which functions as the heterodimeric complex NAE1–UBA3, adenosine sulfamate MLN4924, which is structurally similar to the adenylate intermediate of the NAE catalytic reaction [19]. Early crystal structures of NAE with bound MLN4924 were not particularly informative in terms of explaining the high potency and selectivity of the drug. However, a later crystal structure of the NAE–NEDD8~MLN4924 ternary complex obtained in the presence of NEDD8 and ATP revealed the formation of covalent NEDD8~MLN4924 adduct that blocks the active site of NAE [196] (Figure 12). The selectivity for NAE over other E1s was shown to be achieved via substrate-assisted inhibition [197]. The MLN4924-induced inhibition of NEDD8 transfer leads to an increase in non-NEDDylated CRLs with suppressed ubiquitination activity against their substrates. The compound is now being tested in Phase I clinical trials against a number of cancers [198].

Another approach to target the NEDD8 pathway was undertaken using a 26-mer peptide that corresponds to the NTD of Ubc12, a NEDD8 E2 enzyme [199]. Structural studies revealed that the peptide binds NAE and specifically inhibits the formation of the thioester bond between NEDD8 and Ubc12. The crucial role of NEDD8 in regulation of CRL activity and the tractability of the NEDDylation enzymes for small-molecule modulation together underline the potential of targeting this pathway for future therapeutic intervention.

## CONCLUSIONS

The CRLs are the largest family of multisubunit E3 ligases in humans that are responsible for the recognition, polyubiquitination and degradation of a wide range of substrate proteins. Many members of this family, and of the biological substrates that they regulate, have crucial roles in cellular physiology and homoeostasis, and are also implicated in a wide range of diseases. Disease-linked mutations are increasingly being found in genes that code for either E3 substrates or components of E3 ligase themselves. This, among other compelling evidence, makes CRLs attractive targets; however, conventional drug discovery approaches have largely neglected such large multicomponent cellular machineries because they are traditionally viewed as difficult to target. As a result, the field is still in its infancy and the true potential of this target class has only recently been recognized as bearing adequate risk/gain balance to motivate directing strategic investments and drug discovery efforts into. Nevertheless, increasing understanding of the structural assembly and interactions of these proteins is now emerging which is underpinning growing activity and recent successes at identifying potent cell-active small molecules, particularly using structure-guided approaches. Such high-quality compounds would bind to CRLs, and thus specifically modulate their assembly and/or PPIs, and, as a result, affect their biological function. Given the complexity of CRLs as targets and the different layers of regulation to which they are subject, it is anticipated that small molecules with diverse mechanisms of action should emerge, as not only inhibitors but also activators, not only disruptors but also stabilizers of given PPIs, and modulators of protein dynamics. Pharmacological responses could result from acting directly at specific PPIs or from targeting distant allosteric binding sites. However, few detailed mechanistic, e.g. kinetic and thermodynamic, studies of available CRL ligands have been performed to date. This is possibly because of a paucity of convenient and portable assays for monitoring enzyme activity, and of the difficulty of obtaining many of the protein components and complexes in quantities suitable to detailed biophysical characterization. We believe such mechanistic information to be very important in drug discovery projects, as it can help to maximize chances of success. We therefore predict that significant advancements in these directions are warranted in the near future, which will fuel exciting new developments against a challenging, but potentially highly rewarding, target class.

### Note added in proof (received 17 March 2015)

Since the acceptance of this manuscript, a group from Yale University published the first crystal structure of a quaternary complex between VHL, EloBC and an N-terminal fragment of Cullin2, which is beginning to shine lights into the molecular basis of Cul2 vs Cul5 selectivity by ECS-type CRLs (Nguyen, H.C., Yang, H., Fribourgh, J.L., Wolfe, L.S. and Xiong, Y. Insights into Cullin-RING E3 Ubiquitin Ligase Recruitment: Structure of the VHL-EloBC-Cul2 Complex. Structure 23, 441–449 (2015))

## Online data

Supplementary data
