# Supplementary material for: Targeting Cullin–RING E3 ubiquitin ligases for drug discovery: structure, assembly and small-molecule modulation
Source: Biochem J. 2015 Apr 17;467(Pt 3):365–86. doi: 10.1042/BJ20141450 (PMC4403949; doi:10.1042/BJ20141450)
Supplement: Supplementary data [file bj4670365ntsadd.pdf]

Table S1. Reported X-ray crystal structures of the CRL subunits, their complexes and full-size E3 ligases. Data includes structures with inhibitors and substrate peptides.

PDB code	Protein (Complex)	Components	Resolution, Å	Reference / Year of PDB release	Structure
**CRL1**					
1LDJ<br>1LDK	Cul1-Rbx1	**Cul1** (17-776, Human)<br>**Rbx1** (19-108, Human)	3.00	[1] / 2002	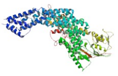
	Cul1-Rbx1-Skp1-Skp2	**Cul1NTD** (15-410, Human)<br>**Cul1CTD** (411-776, Human)<br>**Rbx1** (1-90, Human)<br>**Skp1** (1-133, Human)<br>**Skp2** (1-41, Human)	3.10		
4F52	Glomulin-Cul1-Rbx1	**Glomulin** (1-596, Human)<br>**Cul1** (411-690, Human)<br>**Rbx1** (5-108, Human)	3.00	[2] / 2012	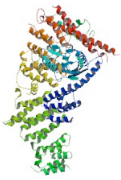
4P5O	Rbx1-Ubc12~NEDD8-Cul1-Dcn1	**Rbx1** (106, Human)<br>**Ubc12** (189, Human)<br>**NEDD8** (81, Human)<br>**Cul1** (368, Human)<br>**Dcn1** (200, Human)	3.11	[3] / 2014	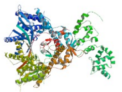
3TDU<br>3TDZ	Cul1-Dcn1-UBE2M	**Cul1** (702-776, Human)<br>**Dcn1** (62-259, Human)<br>**UBE2M** (2-15, Human)	1.50	[4] / 2011	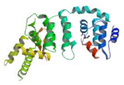
	Cul1-Dcn1-UBE2M	**Cul1** (702-776, Human)<br>**Dcn1** (62-259, Human)<br>**UBE2M** (2-12, Human)	2.0		
3O6B<br>3O2P	Dcn1-Cdc53	**Dcn1** (70-269, S. cerevisiae)<br>**Cdc53** (742-815, S. cerevisiae)	3.1	[5] / 2010	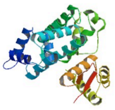
		**Dcn1** (70-269, S. cerevisiae)<br>**Cdc53** (730-815, S. cerevisiae)	2.23		
1U6G	CAND1-Cul1-Rbx1	**Cul1** (1-776, Human)<br>**Rbx1** (1-108, Human)<br>**CAND1** (1-1230, Human)	3.10	[6] / 2004	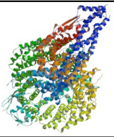
3RTR	Cul1$_{CTD}$-Rbx1	**Cul1$_{CTD}$** (411-776, Human)<br>**Rbx1** (5-108, Human)	3.21	[7] / 2011	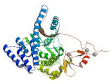
1FQV<br>1FS1<br>1FS2	Skp1-Skp2	**Skp1** (1-149, Human)<br>**Skp2** (101-436, Human)	1.8 – 2.9	[8] / 2000	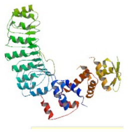
		**Skp1** (1-147, Human)<br>**Skp2** (101-153, Human)			
		**Skp1** (141, Human<br>**Skp2** (272, Human)			
1UMH	Fbxo2	**Fbxo2** (184, Mouse)	2.00	[9] / 2004	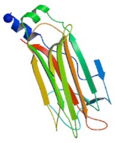

2OVP<br>2OVR<br>2OVQ	Fbw7-Skp1	**Fbw7** (263-707, Human)<br>**Skp1** (1-147, Human)	2.50 – 2.90	[10] / 2007	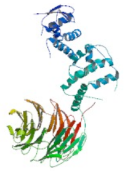
	CyclinE- Fbw7-Skp1	**CyclinE** (8)<br>**Skp1** (1-147, Human)<br>**Fbw7** (263-707, Human)			
	CyclinE- Fbw7-Skp1	**CyclinE** (12)<br>**Fbw7** (263-707, Human)<br>**Skp1** (1-147, Human)			
2ASS<br>2AST	Cks1-Skp2-Skp1	**Cks1** (1-69, Human)<br>**Skp2** (1-336, Human)<br>**Skp1** (1-159, Human)	3.00	[11] / 2005	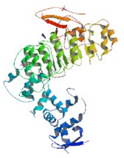
	p27-Cks1-Skp2-Skp1	**p27** (10, Human)<br>**Cks1** (69, Human)<br>**Skp2** (336, Human)<br>**Skp1** (159, Human)	2.3		
1QB3	Cks1	**Cks1** (150, S. cerevisiae)	3.00	[12] / 2000	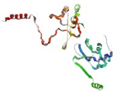
1BUH	Cks1-Cdk2	**Cks1** (79, Human)<br>**Cdk2** (298, Human)	2.60	[13] / 1998	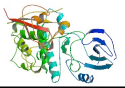
1JSU	p27-Cdk2-Cyclin A	**p27** (22-106, Human)<br>**Cdk2** (298, pT160, Human)<br>**Cyclin A** (173-432, Human)	2.30	[14] / 1997	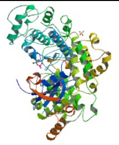
4I6J	Cry2- Fbxl3-Skp1	**Cry2** (544, Mouse)<br>**Fbxl3** (428, Human)<br>**Skp1** (163, Human)	2.70	[15] / 2013	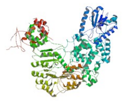
2P1M<br>2P1P<br>2P1Q	Fbl1-Skp1	**Fbl1** (594, Arabidopsis thaliana)<br>**Skp1** (160, Arabidopsis thaliana)	1.80 – 2.21	[16] / 2007	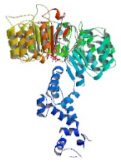
2P1N<br>2P1O	IAA7-Fbl1-Skp1	**IAA7** (13, Arabidopsis thaliana)<br>**Fbl1** (594, Arabidopsis thaliana)<br>**Skp1** (160, Arabidopsis thaliana)	1.90 – 2.50		
2P63	D domain of Cdc4	**Cdc4** (56, S. cerevisiae)	2.67	[17] / 2007	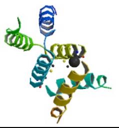
2P64	D domain of βTrCP	**βTrCP** (52, Human)	2.50		
3OGK<br>3OGL<br>3OGM	JAZ1-COI1-Skp1	**JAZ1** (22, Arabidopsis thaliana)<br>**COI1** (592, Arabidopsis thaliana)<br>**Skp1** (160, Arabidopsis thaliana)	2.80 – 3.34	[18] / 2010	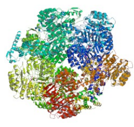
3C6N<br>3C6O<br>3C6P	TIR1-Skp1<br>(+ ligands)	**TIR1** (594, Arabidopsis thaliana)<br>**Skp1** (160, Arabidopsis thaliana)	2.60 – 2.70	[19] / 2008	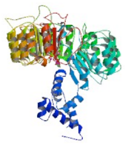

CRL2					
1VCB	VHL-EloB-EloC	**VHL** (54-213, Human)<br>**EloB** (1-120, Human)<br>**EloC** (117-112, Human)	2.70	[20] / 1999	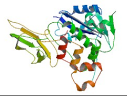
1HV2	VHL-EloC	**VHL** (157-171, Mouse)<br>**EloC** (1-99, S. cerevisiae)	NMR	[21] / 2001	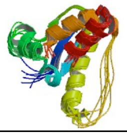
1LQB	HIF-1α-VHL-EloB--EloC	**HIF-1α** (549-582, Human)<br>**VHL** (52-213, Human)<br>**EloB** (118, Human)<br>**EloC** (17-112, Human)	2.00	[22] / 2002	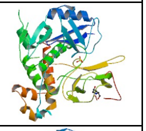
1LM8	HIF-1α-VHL-EloB--EloC	**HIF-1α** (556-575, Human)<br>**VHL** (160, Human)<br>**EloB** (118, Human)<br>**EloC** (96, Human)	1.85	[23] / 2002	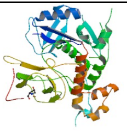
3ZRF<br>3ZTC<br>4B9K<br>4W9H	VHL-EloB-EloC (with ligands)	**VHL** (54-213, Human)<br>**EloB** (1-118, Human)<br>**EloC** (17-112, Human)	2.00 – 2.80	[24-27] / 2012, 2014	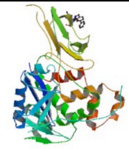

CRL3

4HXI	KLHL3-Cul3	**KLHL3** (1-277, Human)<br>**Cul3** (1-386, Human)	3.51	[28] / 2013	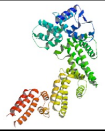
4EOZ	SPOP-Cul3	**SPOP** (177-319, Human)<br>**Cul3** (20-381, Human)	2.40	[29] / 2012	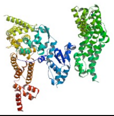
4AP2<br>4APF	KLHL11-Cul3	**KLHL11** (67-340, Human)<br>**Cul3** (1-388, Human)<br>**KLHL11** (67-340, Human)<br>**Cul3** (23-388, Human)	2.80<br>3.1	[30] / 2012	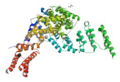
4HS2<br>4J8Z	SPOP	**SPOP** (270-374, Human)<br>**SPOP** (178-374, Human)	1.53<br>2.42	[31] / 2013	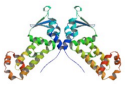
1IUY	Cul3	**Cul3** (1-92, Mouse)	NMR	Inoue, et. al. / 2003	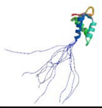
1CS3	ZBTB16	**ZBTB16** (116, Human)	2.00	[32] / 1999	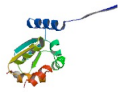
1ZGK	Keap1	**Keap1** (308, Human)	1.35	[33] / 2005	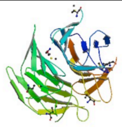
4CHB<br>4CH9	WNK4-KLHL2<br>WNK4-KLHL3	**WNK4** (11, Human)<br>**KLHL2** (302, Human)<br>**WNK4** (11, Human)<br>**KLHL3** (292, Human)	1.56 – 1.84	[34] / 2014	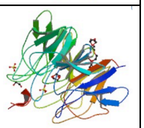
3HQH<br>3HQI<br>3HQL<br>3HQM<br>3HSV<br>3HTM<br>3HU6<br>3HVE<br>3IVQ<br>3IVV<br>3IVB	SPOP-Substrate complex	**SPOP**^{MATHx} (145, Human)<br>**MacroH2A**^{SBCpep1} (15, Human)<br>**SPOP**$^{MATHx-BTB/3-box}$ (312, Human)<br>**Puc**^{SBC1pep3} (7)<br>**SPOP**^{MATHx} (145, Human)<br>**Puc**$^{SBC1\_pep2}$ (16)<br>**SPOP**^{MATHx} (145, Human)<br>**Ci**^{SBC2} (12)<br>**SPOP**^{MATHx} (145, Human)<br>**MacroH2A**^{SBCpep2} (16, Human)<br>**SPOP** (172, Human)<br>**SPOP**$^{MATHx-BTB/3-box}$ (312, Human)<br>**Puc**^{SBC1pep3} (7)<br>**SPOP**$^{BTB/3-box}$ (256, Human)<br>**Gig**$^{BTB/3-box+}$ (254, Human)<br>**SPOP**^{MATH} (145, Human)<br>**Ci**^{SBC2} (12)<br>**SPOP**^{MATH} (145, Human)<br>**Puc**$^{SBC1\_pep1}$ (10)<br>**SPOP**^{MATH} (145, Human)<br>**MacroH2A**^{SBCpep1} (15)	1.25 – 2.80	[35] / 2009	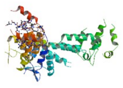

CRL4

4A64	$Cul4B_{NTD}$	**$Cul4B_{NTD}$** (188-539, Human)	2.57	Vollmar, et. al. / 2012	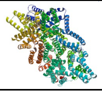
4A0K<br>4A0C<br>4A0L<br>4A11	DNA-DDB2-DDB1--Cul4A-Rbx1	**DDB2** (60-423, Danio rerio)<br>**DDB1** (1159, Human)<br>**Cul4A** (38-759, Human)<br>**Rbx1** (12-108, Mouse)<br>**DNA** (12 bp)	5.93	[36] / 2011	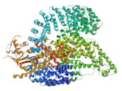
	CAND1-Cul4B-Rbx1	**CAND1** (1253, Human)<br>**Cul4B** (741, Human)<br>**Rbx1** (98, Mouse)	3.80		
4A08<br>4A09<br>4A0A<br>4A0B	DNA-DDB2-DDB1--Cul4B-Rbx1	**DDB2** (60-423, Danio rerio)<br>**DDB1** (1144, Human)<br>**Cul4B** (193-913, Human)<br>**Rbx1** (12-108, Mouse)<br>**DNA** (12 bp)	7.4		
	DDB1-CSA	**DDB1** (1159, Human)<br>**CSA** (408, Human)	3.31		
2HYE	SV5V-DDB1-Cul4A--Rbx1	**SV5V** (1-222, Parainfluenza virus 5)<br>**DDB1** (1-1140, Human)<br>**Cul4A** (1-759, Human)<br>**Rbx1** (1-108, Human)	3.10	[37] / 2006	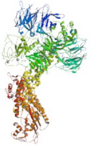
2B5L<br>2B5M<br>2B5N	SV5V-DDB1	**SV5V** (222, Parainfluenza virus 5)<br>**DDB1** (1140, Human)	2.85	[38] / 2006	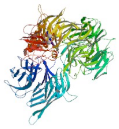
	DDB1	**DDB1** (1140, Human)	2.92		
	$DDB1_{BPB}$	**DDB1** (323, Human)	2.80		
2DO7	Cul4B	**Cul4B** (1-101, Human)	NMR	Suzuki, et. al. / 2007	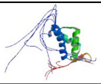
3EI1<br>3EI2<br>3EI3<br>3EI4	DDB1-DDB2	**DDB1** (1158, Human)<br>**DDB2** (383, Danio rerio)	2.30 – 2.80	[39] / 2009	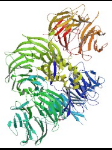
		**DDB1** (1158, Human)<br>**DDB2** (436, Danio rerio)	3.30		
3I7H<br>3I7K<br>3I7N<br>3I7L<br>3I8C<br>3I89<br>3I7O<br>3I8E<br>3I7P	HBx-DDB1	**HBx** (14, Hepatitis B virus)<br>**DDB1** (1-1143, Human)	2.90	[40] / 2009	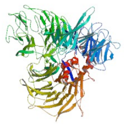
	WHx-DDB1	**WBx (**86-99, Woodchuck hepatitis virus 8)<br>**DDB1** (1-1143, Human)	2.80		
	DCAF9-DDB1	**DCAF9 (**5-17, Human)<br>**DDB1** (1-1143, Human)	2.80		
	DDB2-DDB1	**DDB2 (**68-81, Human)<br>**DDB1** (1-1143, Human)	2.80		
	DCAF4-DDB1	**DCAF4 (**124-136, Human)<br>**DDB1** (1-1143, Human)	2.80		
	DCAF5-DDB1	**DCAF5 (**13, Human)<br>**DDB1** (1-1143, Human)	3.00		
	DCAF6-DDB1	**DCAF6 (**9-21, Human)<br>**DDB1** (1-1143, Human)	2.80		
	DCAF8-DDB1	**DCAF8 (**153-165, Human)<br>**DDB1** (1-1143, Human)	3.40		
	DCAF12-DDB1	**DCAF12 (**45-57, Human)<br>**DDB1** (1-1143, Human)	3.00		
3WA0	Merlin-DCAF1	**Merlin** (301, Mouse)<br>**DCAF1** (96, Human)	2.31	[41] / 2014	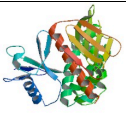

**CRL5**					
4N9F	Vif-CBFβ-Cul5-EloB--EloC	**Cul5** (12-321, Human)<br>**EloC** (1-96, Human)<br>**EloB** (1-102, Human)<br>**CBFβ** (1-170, Human)<br>**Vif** (1-176, HIV-1)	3.30	[42] / 2014	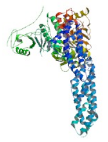
3DCG	Vif-EloB-EloC	**Vif** (139-176, HIV-1)<br>**EloB** (118, Human)<br>**EloC** (17-112, Human)	2.40	[43] / 2008	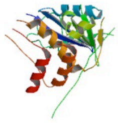
2C9W	SOCS2-EloB-EloC	**SOCS2** (169, Human)<br>**EloB** (118, Human)<br>**EloC** (97, Human)	1.90	[44] / 2006	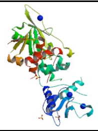
4JGH	SOCS2-EloB-EloC--Cul5$_{NTD}$	**SOCS2** (32-198, Human)<br>**EloB** (2-118, Mouse)<br>**EloC** (17-112, Mouse)<br>**Cul5$_{NTD}$** (10-386, Human)	3.00	[45] / 2013	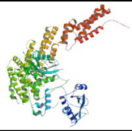
3DPL<br>3DQV	Cul5$_{CTD}$-Rbx1	**Cul5$_{CTD}$** (401-780, Human)<br>**Rbx1** (1-106, Human)	2.60	[46] / 2008	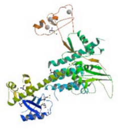
	NEDD8~Cul5$_{CTD}$-Rbx1	**NEDD8** (1-81, Human)<br>**Cul5$_{CTD}$** (401-780, Human)<br>**Rbx1** (5-108, Human)	3.0		
2WZK	Cul5$_{NTD}$	**Cul5$_{NTD}$** (1-386, Mouse)	2.05	[47] / 2009	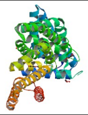
2IZV	SOCS4-EloB-EloC	**SOCS4** (274-437, Human)<br>**EloB** (118, Human)<br>**EloC** (97, Human)	2.55	[48] / 2006	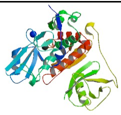
2HMH	GP130-SOCS3	**GP130** (11, Mouse)<br>**SOCS3** (152, Mouse)	2.00	[49] / 2006	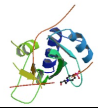
4GL9	GP130- JAK2-SOCS3	**GP130** (15, Mouse)<br>**JAK2** (297, Mouse)<br>**SOCS3** (143, Mouse)	3.90	[50] / 2013	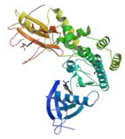
2BBU	GP130-SOCS3	**GP130** (15, Mouse)<br>**SOCS3** (164, Mouse)	NMR	[51] / 2006	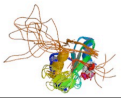
2VIF	cKIT-SOCS6$_{SH2}$	**cKIT** (564-574, Human)<br>**SOCS6** (361-499, Human)	1.45	[52] / 2007	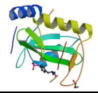
3ZKJ	ASB9-EloB-EloC	**ASB9** (261, Human)<br>**EloB** (118, Human)<br>**EloC** (96, Human)	2.58	[47] / 2013	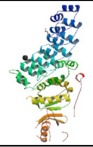
3ZNG	ASB9-EloB-EloC	**ASB9** (268, Human)<br>**EloB** (118, Human)<br>**EloC** (97, Human)	2.85	[53] / 2013	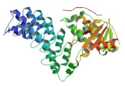
**CRL7**					
2JNG	Cul7	**Cul7** (360-460, Human)	NMR	[54] / 2007	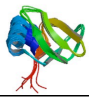

Structures with bound inhibitors

3RZ3	Cdc34-CC0651	**Cdc34 (E2)** (7-184, Human)<br>**CC0651** ($C_{20}H_{21}Cl_2NO_6$)	2.30		
4MDK	Ubiquitin-Cdc34-CC0651	**Ubiquitin** (80, Human)<br>**Cdc34 (E2)** (7-184, Human)<br>**CC0651** ($C_{20}H_{21}Cl_2NO_6$)	2.61	[55] / 2013	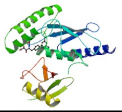
3MKS	Cdc4-Skp1 in complex with allosteric inhibitor SCF-I2	**Skp1** (2-[37-64]-194, S. cerevisiae)<br>**Cdc4** (263-[602-605, 609-624]-744, S. cerevisiae)<br>**SCF-I2** ($C_{22}H_{14}O_4$)	2.60	[56] / 2010	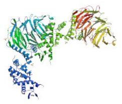
3GZN	NAE1-UBA3-NEDD8-MLN4924	**NAE1** (534, Human)<br>**UBA3** (463, Human)<br>**NEDD8** (82, Human)<br>**MLN4924** ($C_{21}H_{25}N_5O_4S$)	3.00	[57] / 2010	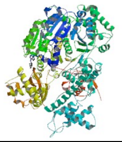
3C6N	TIR1-Skp1 in complex with Probe 8	**TIR1** (594, Arabidopsis thaliana)<br>**Skp1** (160, Arabidopsis thaliana)<br>**Probe 8** ($C_{21}H_{30}N_2O_4$)	2.60	[19] / 2008	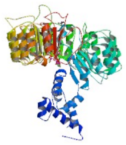
3ZRF<br>3ZTC<br>4B9K<br>4W9H	VHL-EloB-EloC in complex with Compound 7 / Compound 15	**VHL** (54-213, Human)<br>**EloB** (1-118, Human)<br>**EloC** (17-112, Human)<br>**Compound 7** ($C_{24}H_{32}N_4O_4S$)<br>**Compound 15** ($C_{21}H_{22}N_4O_5$)	2.00 – 2.80	[24-27] / 2012, 2014	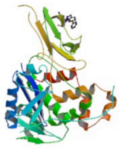
4CI1<br>4CI2<br>4CI3	CRBN-DDB1 in complex with Thalidomide / Lenalidomide / Pomalidomide	**CRBN** (469, G. gallus)<br>**DDB1** (1158, Human)<br>**Thalidomide** ($C_{13}H_{10}N_2O_4$) /<br>**Lenalidomide** ($C_{13}H_{13}N_3O_3$) /<br>**Pomalidomide** ($C_{13}H_{11}N_3O_4$)	2.95 – 3.50	[58] / 2014	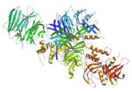
4TZ4<br>4TZU<br>4TZC<br>3WX2	CRBN-DDB1 in complex with Lenalidomide	**CRBN** (381, Human)<br>**DDB1** (1146, Human)<br>**Lenalidomide** ($C_{13}H_{13}N_3O_3$)	1.88 – 3.01	[59] / 2014	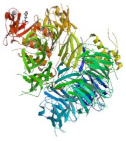
	CRBN in complex with Thalidomide / Pomalidomide	**CRBN** (108, Mouse)<br>**Thalidomide** ($C_{13}H_{10}N_2O_4$) /<br>**Pomalidomide** ($C_{13}H_{11}N_3O_4$)			
4L7B<br>4L7C<br>4N1B<br>4L7D	Keap1 with SRS-1a / SRS-22 / SRS-43 / SRS-59	**Keap1** (300, Human)<br>**SRS-1a** ($C_{26}H_{26}N_2O_5$)<br>**SRS-22** ($C_{26}H_{26}N_6O_3$)<br>**SRS-43** ($C_{26}H_{28}N_2O_4$)<br>**SRS-59** ($C_{27}H_{30}N_2O_4$)	2.25 – 2.55	[60] / 2014	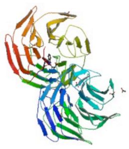
4IN4<br>4IQK	Keap1 with Cpd15 / Cpd16	**Keap1** (321-609, Human)<br>**Cpd15** ($C_{21}H_{18}F_3N_3O_4S_2$)<br>**Cpd16** ($C_{24}H_{22}N_2O_6S_2$)	1.97 – 2.59	[61] / 2013	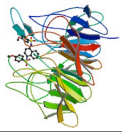

Structures with bound substrate peptides					
2AST	p27-Cks1-Skp2-Skp1	**p27** (10, Human)<br>**Cks1** (69, Human)<br>**Skp2** (336, Human)<br>**Skp1** (159, Human)	2.3	[11] / 2005	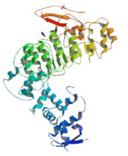
2P1N<br>2P1O	IAA7-Fbl1-Skp1	**IAA7** (13, Arabidopsis thaliana)<br>**Fbl1** (594, Arabidopsis thaliana)<br>**Skp1** (160, Arabidopsis thaliana)	1.90 – 2.50	[16] / 2007	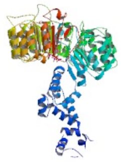
3OGK<br>3OGL<br>3OGM	JAZ1-COI1-Skp1	**JAZ1** (22, Arabidopsis thaliana)<br>**COI1** (592, Arabidopsis thaliana)<br>**Skp1** (160, Arabidopsis thaliana)	2.80 – 3.34	[18] / 2010	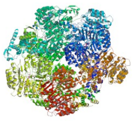
1NEX	CPD-Cdc4-Skp1	**CPD** (9, S. cerevisiae**)**<br>**Cdc4** (1-[601-604, 609-624]-779, S. cerevisiae)<br>**Skp1** (1-[36-63]-194, S. cerevisiae)	2.7	[62] / 2003	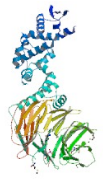
3V7D	Sic1-Cdc4-Skp1	**Sic1** (67-85, S. cerevisiae)<br>**Cdc4** (263-744, S. cerevisiae)<br>**Skp1** (169, S. cerevisiae)	2.31	[63] / 2011	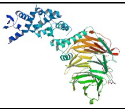
1P22	β-catenin-βTrCP1-Skp1	**β-Catenin** (1-26, Human)<br>**βTrCP1** (1-435, Human)<br>**Skp1** (1-145, Human)	2.95	[64] / 2003	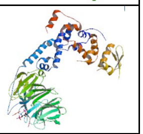
4LPA	Cks1 with Phosphothreonine	**Cks1** (1-113, S. cerevisiae)<br>Phospho-threonine	2.90	[65] / 2013	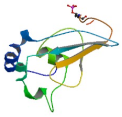
2FLU	Nrf2-Keap1	**Nrf2** (16, Human)<br>**Keap1** (308, Human)	1.50	[66] / 2006	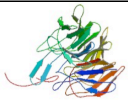
2DYH	Nrf2-Keap1	**Nrf2** (15, Human)<br>**Keap1** (318, Human)	1.90	[67] / 2007	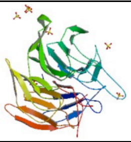
1LQB	HIF1α -VHL-EloB-EloC	**HIF-1α** (549-582, Human)<br>**VHL** (52-213, Human)<br>**EloB** (118, Human)<br>**EloC** (17-112, Human)	2.00	[22] / 2002	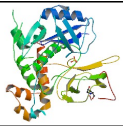
1LM8	HIF1α-VHL-EloB-EloC-	**HIF-1α** (556-575, Human)<br>**VHL** (160, Human)<br>**EloB** (118, Human)<br>**EloC** (96, Human)	1.85	[23] / 2002	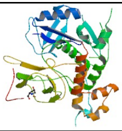
2VIF	cKIT-SOCS6$_{SH2}$-	**cKIT** (564-574, Human)<br>**SOCS6$_{SH2}$** (361-499, Human)	1.45	[52] / 2007	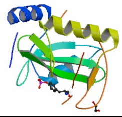
2HMH	GP130-SOCS3	**GP130** (11, Mouse)<br>**SOCS3** (152, Mouse)	2.00	[49] / 2006	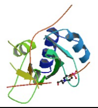

Other relevant structures					
2LGV	Rbx1	**Rbx1** (1-100, Human)	NMR	[68] / 2012	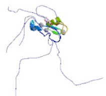
4F7O	CSN5	**CSN5** (1-275, Human)	2.6	[69] / 2013	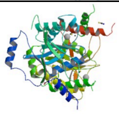
2JUF	$PARC_{CPH}$	**PARC** (366-466, Human)	NMR	Kaustov, et. al. / 2007	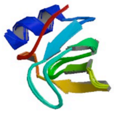
1NDD	NEDD8	**NEDD8** (76, Human)	1.60	[70] / 1999	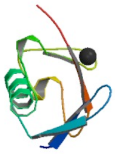
1R4N<br>1R4M	NAE1-UBA3-NEDD8-ATP	**NAE1** (529, Human)<br>**UBA3** (431, Human)<br>**NEDD8** (76, Human)	3.60	[71] / 2003	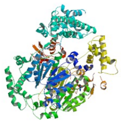
	NAE1-UBA3-NEDD8	**NAE1** (529, Human)<br>**UBA3** (431, Human)<br>**NEDD8** (76, Human)	3.00		
1TT5	NAE1-UBA3-UBE2M (NEDD8 E1 + NEDD8 E2 peptide)	**NAE1** (531, Human)<br>**UBA3** (434, Human)<br>**UBE2M** (26, Human)	2.60	[72] / 2004	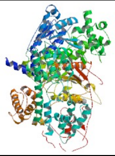
4D18<br>4D10	CSN	**COP9**<br>**Subunit 1** (480, Human)<br>**Subunit 2** (447, Human)<br>**Subunit 3** (427, Human)<br>**Subunit 4** (410, Human)<br>**Subunit 5** (327, Human)<br>**Subunit 6** (331, Human)<br>**Subunit 7A** (222, Human)<br>**Subunit 8** (213, Human)	3.80 – 4.08	[73] / 2014	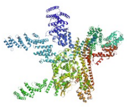

1 Zheng, N., Schulman, B. A., Song, L., Miller, J. J., Jeffrey, P. D., Wang, P., Chu, C., Koepp, D. M., Elledge, S. J., Pagano, M., et al. (2002) Structure of the Cul1-Rbx1-Skp1-F boxSkp2 SCF ubiquitin ligase complex. Nature **416**, 703–709.
2 Duda, D. M., Olszewski, J. L., Tron, A. E., Hammel, M., Lambert, L. J., Waddell, M. B., Mittag, T., DeCaprio, J. A. and Schulman, B. A. (2012) Structure of a glomulin-RBX1-CUL1 complex: inhibition of a RING E3 ligase through masking of its E2-binding surface. Mol. Cell **47**, 371–382.
3 Scott, D. C., Sviderskiy, V. O., Monda, J. K., Lydeard, J. R., Cho, S. E., Harper, J. W. and Schulman, B. A. (2014) Structure of a RING E3 trapped in action reveals ligation mechanism for the ubiquitin-like protein NEDD8. Cell **157**, 1671–1684.
4 Scott, D. C., Monda, J. K., Bennett, E. J., Harper, J. W. and Schulman, B. A. (2011) N-terminal acetylation acts as an avidity enhancer within an interconnected multiprotein complex. Science **334**, 674–678.
5 Scott, D. C., Monda, J. K., Grace, C. R. R., Duda, D. M., Kriwacki, R. W., Kurz, T. and Schulman, B. A. (2010) A dual E3 mechanism for Rub1 ligation to Cdc53. Mol. Cell **39**, 784–796.
6 Goldenberg, S. J., Cascio, T. C., Shumway, S. D., Garbutt, K. C., Liu, J., Xiong, Y. and Zheng, N. (2004) Structure of the Cand1-Cul1-Roc1 complex reveals regulatory mechanisms for the assembly of the multisubunit cullin-dependent ubiquitin ligases. Cell **119**, 517–528.
7 Calabrese, M. F., Scott, D. C., Duda, D. M., Grace, C. R. R., Kurinov, I., Kriwacki, R. W. and Schulman, B. A. (2011) A RING E3-substrate complex poised for ubiquitin-like protein transfer: structural insights into cullin-RING ligases. Nat. Struct. Mol. Biol. **18**, 947–949.
8 Schulman, B. A., Carrano, A. C., Jeffrey, P. D., Bowen, Z., Kinnucan, E. R., Finnin, M. S., Elledge, S. J., Harper, J. W., Pagano, M. and Pavletich, N. P. (2000) Insights into SCF ubiquitin ligases from the structure of the Skp1-Skp2 complex. Nature **408**, 381–386.
9 Mizushima, T., Hirao, T., Yoshida, Y., Lee, S. J., Chiba, T., Iwai, K., Yamaguchi, Y., Kato, K., Tsukihara, T. and Tanaka, K. (2004) Structural basis of sugar-recognizing ubiquitin ligase. Nat. Struct. Mol. Biol. **11**, 365–370.
10 Hao, B., Oehlmann, S., Sowa, M. E., Harper, J. W. and Pavletich, N. P. (2007) Structure of a Fbw7-Skp1-cyclin E complex: multisite-phosphorylated substrate recognition by SCF ubiquitin ligases. Mol. Cell **26**, 131–143.
11 Hao, B., Zheng, N., Schulman, B. A., Wu, G., Miller, J. J., Pagano, M. and Pavletich, N. P. (2005) Structural basis of the Cks1-dependent recognition of p27(Kip1) by the SCF(Skp2) ubiquitin ligase. Mol. Cell **20**, 9–19.
12 Bourne, Y., Watson, M. H., Arvai, A. S., Bernstein, S. L., Reed, S. I. and Tainer, J. A. (2000) Crystal structure and mutational analysis of the Saccharomyces cerevisiae cell cycle regulatory protein Cks1: implications for domain swapping, anion binding and protein interactions. Structure **8**, 841–850.
13 Bourne, Y., Watson, M. H., Hickey, M. J., Holmes, W., Rocque, W., Reed, S. I. and Tainer, J. A. (1996) Crystal structure and mutational analysis of the human CDK2 kinase complex with cell cycle-regulatory protein CksHs1. Cell **84**, 863–874.
14 Russo, A. A., Jeffrey, P. D., Patten, A. K., Massagué, J. and Pavletich, N. P. (1996) Crystal structure of the p27Kip1 cyclin-dependent-kinase inhibitor bound to the cyclin A-Cdk2 complex. Nature **382**, 325–331.

15 Xing, W., Busino, L., Hinds, T. R., Marionni, S. T., Saifee, N. H., Bush, M. F., Pagano, M. and Zheng, N. (2013) SCF(FBXL3) ubiquitin ligase targets cryptochromes at their cofactor pocket. Nature **496**, 64–68.

16 Tan, X., Calderon-Villalobos, L. I. A., Sharon, M., Zheng, C., Robinson, C. V., Estelle, M. and Zheng, N. (2007) Mechanism of auxin perception by the TIR1 ubiquitin ligase. Nature **446**, 640–645.

17 Tang, X., Orlicky, S., Lin, Z., Willems, A., Neculai, D., Ceccarelli, D., Mercurio, F., Shilton, B. H., Sicheri, F. and Tyers, M. (2007) Suprafacial orientation of the SCFCdc4 dimer accommodates multiple geometries for substrate ubiquitination. Cell **129**, 1165–1176.

18 Sheard, L. B., Tan, X., Mao, H., Withers, J., Ben-Nissan, G., Hinds, T. R., Kobayashi, Y., Hsu, F.-F., Sharon, M., Browse, J., et al. (2010) Jasmonate perception by inositol-phosphate-potentiated COI1-JAZ co-receptor. Nature **468**, 400–405.

19 Hayashi, K.-I., Tan, X., Zheng, N., Hatate, T., Kimura, Y., Kepinski, S. and Nozaki, H. (2008) Small-molecule agonists and antagonists of F-box protein-substrate interactions in auxin perception and signaling. PNAS **105**, 5632–5637.

20 Stebbins, C. E., Kaelin, W. G. and Pavletich, N. P. (1999) Structure of the VHL-ElonginC-ElonginB complex: implications for VHL tumor suppressor function. Science **284**, 455–461.

21 Botuyan, M. V., Mer, G., Yi, G. S., Koth, C. M., Case, D. A., Edwards, A. M., Chazin, W. J. and Arrowsmith, C. H. (2001) Solution structure and dynamics of yeast elongin C in complex with a von Hippel-Lindau peptide. J. Mol. Biol. **312**, 177–186.

22 Hon, W.-C., Wilson, M. I., Harlos, K., Claridge, T. D. W., Schofield, C. J., Pugh, C. W., Maxwell, P. H., Ratcliffe, P. J., Stuart, D. I. and Jones, E. Y. (2002) Structural basis for the recognition of hydroxyproline in HIF-1 alpha by pVHL. Nature **417**, 975–978.

23 Min, J.-H., Yang, H., Ivan, M., Gertler, F., Kaelin, W. G. and Pavletich, N. P. (2002) Structure of an HIF-1alpha -pVHL complex: hydroxyproline recognition in signaling. Science **296**, 1886–1889.

24 Buckley, D. L., Van Molle, I., Gareiss, P. C., Tae, H. S., Michel, J., Noblin, D. J., Jorgensen, W. L., Ciulli, A. and Crews, C. M. (2012) Targeting the von Hippel-Lindau E3 ubiquitin ligase using small molecules to disrupt the VHL/HIF-1α interaction. J. Am. Chem. Soc. **134**, 4465–4468.

25 Van Molle, I., Thomann, A., Buckley, D. L., So, E. C., Lang, S., Crews, C. M. and Ciulli, A. (2012) Dissecting fragment-based lead discovery at the von Hippel-Lindau protein:hypoxia inducible factor 1α protein-protein interface. Chem. Biol. **19**, 1300–1312.

26 Buckley, D. L., Gustafson, J. L., Van Molle, I., Roth, A. G., Tae, H. S., Gareiss, P. C., Jorgensen, W. L., Ciulli, A. and Crews, C. M. (2012) Small-molecule inhibitors of the interaction between the E3 ligase VHL and HIF1α. Angew. Chem. Int. Ed. Engl. **51**, 11463–11467.

27 Galdeano, C., Gadd, M. S., Soares, P., Scaffidi, S., Van Molle, I., Birced, I., Hewitt, S., Dias, D. M. and Ciulli, A. (2014) Structure-Guided Design and Optimization of Small Molecules Targeting the Protein-Protein Interaction between the von Hippel-Lindau (VHL) E3 Ubiquitin Ligase and the Hypoxia Inducible Factor (HIF) Alpha Subunit with in Vitro Nanomolar Affinities. J. Med. Chem. **57**, 8657–8663.

28 Ji, A. X. and Privé, G. G. (2013) Crystal structure of KLHL3 in complex with

Cullin3. PLoS ONE **8**, e60445.
29 Errington, W. J., Khan, M. Q., Bueler, S. A., Rubinstein, J. L., Chakrabartty, A. and Privé, G. G. (2012) Adaptor protein self-assembly drives the control of a cullin-RING ubiquitin ligase. Structure **20**, 1141–1153.
30 Canning, P., Cooper, C. D. O., Krojer, T., Murray, J. W., Pike, A. C. W., Chaikuad, A., Keates, T., Thangaratnarajah, C., Hojzan, V., Ayinampudi, V., et al. (2013) Structural basis for Cul3 protein assembly with the BTB-Kelch family of E3 ubiquitin ligases. J. Biol. Chem. **288**, 7803–7814.
31 van Geersdaele, L. K., Stead, M. A., Harrison, C. M., Carr, S. B., Close, H. J., Rosbrook, G. O., Connell, S. D. and Wright, S. C. (2013) Structural basis of high-order oligomerization of the cullin-3 adaptor SPOP. Acta Cryst. D **69**, 1677–1684.
32 Li, X., Peng, H., Schultz, D. C., Lopez-Guisa, J. M., Rauscher, F. J. and Marmorstein, R. (1999) Structure-function studies of the BTB/POZ transcriptional repression domain from the promyelocytic leukemia zinc finger oncoprotein. Cancer Res. **59**, 5275–5282.
33 Beamer, L. J., Li, X., Bottoms, C. A. and Hannink, M. (2005) Conserved solvent and side-chain interactions in the 1.35 Angstrom structure of the Kelch domain of Keap1. Acta Cryst. D **61**, 1335–1342.
34 Schumacher, F.-R., Sorrell, F. J., Alessi, D. R., Bullock, A. N. and Kurz, T. (2014) Structural and biochemical characterization of the KLHL3-WNK kinase interaction important in blood pressure regulation. Biochem. J. **460**, 237–246.
35 Zhuang, M., Calabrese, M. F., Liu, J., Waddell, M. B., Nourse, A., Hammel, M., Miller, D. J., Walden, H., Duda, D. M., Seyedin, S. N., et al. (2009) Structures of SPOP-substrate complexes: insights into molecular architectures of BTB-Cul3 ubiquitin ligases. Mol. Cell **36**, 39–50.
36 Fischer, E. S., Scrima, A., Böhm, K., Matsumoto, S., Lingaraju, G. M., Faty, M., Yasuda, T., Cavadini, S., Wakasugi, M., Hanaoka, F., et al. (2011) The molecular basis of CRL4DDB2/CSA ubiquitin ligase architecture, targeting, and activation. Cell **147**, 1024–1039.
37 Angers, S., Li, T., Yi, X., MacCoss, M. J., Moon, R. T. and Zheng, N. (2006) Molecular architecture and assembly of the DDB1-CUL4A ubiquitin ligase machinery. Nature **443**, 590–593.
38 Li, T., Chen, X., Garbutt, K. C., Zhou, P. and Zheng, N. (2006) Structure of DDB1 in complex with a paramyxovirus V protein: viral hijack of a propeller cluster in ubiquitin ligase. Cell **124**, 105–117.
39 Scrima, A., Konícková, R., Czyzewski, B. K., Kawasaki, Y., Jeffrey, P. D., Groisman, R., Nakatani, Y., Iwai, S., Pavletich, N. P. and Thomä, N. H. (2008) Structural basis of UV DNA-damage recognition by the DDB1-DDB2 complex. Cell **135**, 1213–1223.
40 Li, T., Robert, E. I., van Breugel, P. C., Strubin, M. and Zheng, N. (2010) A promiscuous alpha-helical motif anchors viral hijackers and substrate receptors to the CUL4-DDB1 ubiquitin ligase machinery. Nat. Struct. Mol. Biol. **17**, 105–111.
41 Mori, T., Gotoh, S., Shirakawa, M. and Hakoshima, T. (2014) Structural basis of DDB1-and-Cullin 4-associated Factor 1 (DCAF1) recognition by merlin/NF2 and its implication in tumorigenesis by CD44-mediated inhibition of merlin suppression of DCAF1 function. Genes to Cells **19**, 603–619.
42 Guo, Y., Dong, L., Qiu, X., Wang, Y., Zhang, B., Liu, H., Yu, Y., Zang, Y., Yang, M. and Huang, Z. (2014) Structural basis for hijacking CBF-β and CUL5

E3 ligase complex by HIV-1 Vif. Nature **505**, 229–233.
43 Stanley, B. J., Ehrlich, E. S., Short, L., Yu, Y., Xiao, Z., Yu, X.-F. and Xiong, Y. (2008) Structural insight into the human immunodeficiency virus Vif SOCS box and its role in human E3 ubiquitin ligase assembly. J. Virol. **82**, 8656–8663.
44 Bullock, A. N., Debreczeni, J. É., Edwards, A. M., Sundström, M. and Knapp, S. (2006) Crystal structure of the SOCS2-elongin C-elongin B complex defines a prototypical SOCS box ubiquitin ligase. PNAS **103**, 7637–7642.
45 Kim, Y. K., Kwak, M.-J., Ku, B., Suh, H.-Y., Joo, K., Lee, J., Jung, J. U. and Oh, B.-H. (2013) Structural basis of intersubunit recognition in elongin BC-cullin 5-SOCS box ubiquitin-protein ligase complexes. Acta Cryst. D **69**, 1587–1597.
46 Duda, D. M., Borg, L. A., Scott, D. C., Hunt, H. W., Hammel, M. and Schulman, B. A. (2008) Structural insights into NEDD8 activation of cullin-RING ligases: conformational control of conjugation. Cell **134**, 995–1006.
47 Muniz, J. R. C., Guo, K., Kershaw, N. J., Ayinampudi, V., Delft, von, F., Babon, J. J. and Bullock, A. N. (2013) Molecular Architecture of the Ankyrin SOCS Box Family of Cul5-Dependent E3 Ubiquitin Ligases. J. Mol. Biol. **425**, 3166–3177.
48 Bullock, A. N., Rodriguez, M. C., Debreczeni, J. É., Songyang, Z. and Knapp, S. (2007) Structure of the SOCS4-ElonginB/C complex reveals a distinct SOCS box interface and the molecular basis for SOCS-dependent EGFR degradation. Structure **15**, 1493–1504.
49 Bergamin, E., Wu, J. and Hubbard, S. R. (2006) Structural basis for phosphotyrosine recognition by suppressor of cytokine signaling-3. Structure **14**, 1285–1292.
50 Kershaw, N. J., Murphy, J. M., Liau, N. P. D., Varghese, L. N., Laktyushin, A., Whitlock, E. L., Lucet, I. S., Nicola, N. A. and Babon, J. J. (2013) SOCS3 binds specific receptor-JAK complexes to control cytokine signaling by direct kinase inhibition. Nat. Struct. Mol. Biol. **20**, 469–476.
51 Babon, J. J., McManus, E. J., Yao, S., DeSouza, D. P., Mielke, L. A., Sprigg, N. S., Willson, T. A., Hilton, D. J., Nicola, N. A., Baca, M., et al. (2006) The structure of SOCS3 reveals the basis of the extended SH2 domain function and identifies an unstructured insertion that regulates stability. Mol. Cell **22**, 205–216.
52 Zadjali, F., Pike, A. C. W., Vesterlund, M., Sun, J., Wu, C., Li, S. S. C., Rönnstrand, L., Knapp, S., Bullock, A. N. and Flores-Morales, A. (2011) Structural basis for c-KIT inhibition by the suppressor of cytokine signaling 6 (SOCS6) ubiquitin ligase. J. Biol. Chem. **286**, 480–490.
53 Thomas, J. C., Matak-Vinkovic, D., Van Molle, I. and Ciulli, A. (2013) Multimeric complexes among ankyrin-repeat and SOCS-box protein 9 (ASB9), ElonginBC, and Cullin 5: insights into the structure and assembly of ECS-type Cullin-RING E3 ubiquitin ligases. Biochemistry **52**, 5236–5246.
54 Kaustov, L., Lukin, J., Lemak, A., Duan, S., Ho, M., Doherty, R., Penn, L. Z. and Arrowsmith, C. H. (2007) The conserved CPH domains of Cul7 and PARC are protein-protein interaction modules that bind the tetramerization domain of p53. J. Biol. Chem. **282**, 11300–11307.
55 Huang, H., Ceccarelli, D. F., Orlicky, S., St-Cyr, D. J., Ziemba, A., Garg, P., Plamondon, S., Auer, M., Sidhu, S., Marinier, A., et al. (2014) E2 enzyme inhibition by stabilization of a low-affinity interface with ubiquitin. Nat. Chem. Biol. **10**, 156–163.

56 Orlicky, S., Tang, X., Neduva, V., Elowe, N., Brown, E. D., Sicheri, F. and Tyers, M. (2010) An allosteric inhibitor of substrate recognition by the SCF(Cdc4) ubiquitin ligase. Nat. Biotechnol. **28**, 733–737.
57 Brownell, J. E., Sintchak, M. D., Gavin, J. M., Liao, H., Bruzzese, F. J., Bump, N. J., Soucy, T. A., Milhollen, M. A., Yang, X., Burkhardt, A. L., et al. (2010) Substrate-assisted inhibition of ubiquitin-like protein-activating enzymes: the NEDD8 E1 inhibitor MLN4924 forms a NEDD8-AMP mimetic in situ. Mol. Cell **37**, 102–111.
58 Fischer, E. S., Böhm, K., Lydeard, J. R., Yang, H., Stadler, M. B., Cavadini, S., Nagel, J., Serluca, F., Acker, V., Lingaraju, G. M., et al. (2014) Structure of the DDB1-CRBN E3 ubiquitin ligase in complex with thalidomide. Nature **512**, 49–53.
59 Chamberlain, P. P., Lopez-Girona, A., Miller, K., Carmel, G., Pagarigan, B., Chie-Leon, B., Rychak, E., Corral, L. G., Ren, Y. J., Wang, M., et al. (2014) Structure of the human Cereblon-DDB1-lenalidomide complex reveals basis for responsiveness to thalidomide analogs. Nat. Struct. Mol. Biol.
60 Jnoff, E., Albrecht, C., Barker, J. J., Barker, O., Beaumont, E., Bromidge, S., Brookfield, F., Brooks, M., Bubert, C., Ceska, T., et al. (2014) Binding mode and structure-activity relationships around direct inhibitors of the Nrf2-Keap1 complex. ChemMedChem **9**, 699–705.
61 Marcotte, D., Zeng, W., Hus, J.-C., McKenzie, A., Hession, C., Jin, P., Bergeron, C., Lugovskoy, A., Enyedy, I., Cuervo, H., et al. (2013) Small molecules inhibit the interaction of Nrf2 and the Keap1 Kelch domain through a non-covalent mechanism. Bioorg. Med. Chem. **21**, 4011–4019.
62 Orlicky, S., Tang, X., Willems, A., Tyers, M. and Sicheri, F. (2003) Structural basis for phosphodependent substrate selection and orientation by the SCFCdc4 ubiquitin ligase. Cell **112**, 243–256.
63 Tang, X., Orlicky, S., Mittag, T., Csizmok, V., Pawson, T., Forman-Kay, J. D., Sicheri, F. and Tyers, M. (2012) Composite low affinity interactions dictate recognition of the cyclin-dependent kinase inhibitor Sic1 by the SCFCdc4 ubiquitin ligase. PNAS **109**, 3287–3292.
64 Wu, G., Xu, G., Schulman, B. A., Jeffrey, P. D., Harper, J. W. and Pavletich, N. P. (2003) Structure of a beta-TrCP1-Skp1-beta-catenin complex: destruction motif binding and lysine specificity of the SCF(beta-TrCP1) ubiquitin ligase. Mol. Cell **11**, 1445–1456.
65 McGrath, D. A., Balog, E. R. M., Kõivomägi, M., Lucena, R., Mai, M. V., Hirschi, A., Kellogg, D. R., Loog, M. and Rubin, S. M. (2013) Cks confers specificity to phosphorylation-dependent CDK signaling pathways. Nat. Struct. Mol. Biol. **20**, 1407–1414.
66 Lo, S.-C., Li, X., Henzl, M. T., Beamer, L. J. and Hannink, M. (2006) Structure of the Keap1:Nrf2 interface provides mechanistic insight into Nrf2 signaling. EMBO J. **25**, 3605–3617.
67 Tong, K. I., Padmanabhan, B., Kobayashi, A., Shang, C., Hirotsu, Y., Yokoyama, S. and Yamamoto, M. (2007) Different electrostatic potentials define ETGE and DLG motifs as hinge and latch in oxidative stress response. Mol. Cell. Biol. **27**, 7511–7521.
68 Spratt, D. E., Wu, K., Kovacev, J., Pan, Z.-Q. and Shaw, G. S. (2012) Selective recruitment of an E2~ubiquitin complex by an E3 ubiquitin ligase. J. Biol. Chem. **287**, 17374–17385.
69 Echalier, A., Pan, Y., Birol, M., Tavernier, N., Pintard, L., Hoh, F., Ebel, C.,

Galophe, N., Claret, F. X. and Dumas, C. (2013) Insights into the regulation of the human COP9 signalosome catalytic subunit, CSN5/Jab1. PNAS **110**, 1273–1278.

70 Whitby, F. G., Xia, G., Pickart, C. M. and Hill, C. P. (1998) Crystal structure of the human ubiquitin-like protein NEDD8 and interactions with ubiquitin pathway enzymes. J. Biol. Chem. **273**, 34983–34991.

71 Walden, H., Podgorski, M. S., Huang, D. T., Miller, D. W., Howard, R. J., Minor, D. L., Holton, J. M. and Schulman, B. A. (2003) The structure of the APPBP1-UBA3-NEDD8-ATP complex reveals the basis for selective ubiquitin-like protein activation by an E1. Mol. Cell **12**, 1427–1437.

72 Huang, D. T., Miller, D. W., Mathew, R., Cassell, R., Holton, J. M., Roussel, M. F. and Schulman, B. A. (2004) A unique E1-E2 interaction required for optimal conjugation of the ubiquitin-like protein NEDD8. Nat. Struct. Mol. Biol. **11**, 927–935.

73 Lingaraju, G. M., Bunker, R. D., Cavadini, S., Hess, D., Hassiepen, U., Renatus, M., Fischer, E. S. and Thomä, N. H. (2014) Crystal structure of the human COP9 signalosome. Nature **512**, 161–165.
